# Glia fuel neurons with locally synthesized ketone bodies to sustain memory under starvation

**DOI:** 10.1038/s42255-022-00528-6

**Published:** 2022-02-17

**Authors:** Bryon Silva, Olivier L. Mantha, Johann Schor, Alberto Pascual, Pierre-Yves Plaçais, Alice Pavlowsky, Thomas Preat

**Affiliations:** 1 Energy & Memory, Brain Plasticity Unit, CNRS, ESPCI Paris, PSL Research University, Paris, France; 2 Instituto de Biomedicina de Sevilla, Hospital Universitario Virgen del Rocío/CSIC/Universidad de Sevilla, Sevilla, Spain; 3 Present Address: INSERM UMR1069 ‘Nutrition, Croissance et Cancer’, Tours, France

**Keywords:** Long-term memory, Astrocyte, Metabolism

## Abstract

During starvation, mammalian brains can adapt their metabolism, switching from glucose to alternative peripheral fuel sources. In the *Drosophila* starved brain, memory formation is subject to adaptative plasticity, but whether this adaptive plasticity relies on metabolic adaptation remains unclear. Here we show that during starvation, neurons of the fly olfactory memory centre import and use ketone bodies (KBs) as an energy substrate to sustain aversive memory formation. We identify local providers within the brain, the cortex glia, that use their own lipid store to synthesize KBs before exporting them to neurons via monocarboxylate transporters. Finally, we show that the master energy sensor AMP-activated protein kinase regulates both lipid mobilization and KB export in cortex glia. Our data provide a general schema of the metabolic interactions within the brain to support memory when glucose is scarce.

## Abstract (web summary)

In the *Drosophila* starved brain, memory formation undergoes adaptive plasticity. Silva et al. show that neurons in the olfactory memory centre of the starved fly are fuelled by glial-derived ketone bodies in order to sustain memory formation.

## Main

The main energy source for the brain is glucose^1^. Metabolic communication between neurons and glia is crucial to sustain brain functions such as memory^2^. The main model of this metabolic communication is the astrocyte–neuron lactate shuttle (ANLS), wherein glia take up glucose from blood and provide lactate via glycolysis to neurons as an energy substrate; this lactate production is stimulated by neuronal activity^3^. But how is the brain’s energy requirement met during starvation when glucose is scarce? It has been known since the 1960s that, under starvation, the two principal KBs, acetoacetate and β-hydroxybutyrate, are used by the brain to support its energy demand^4^. Nevertheless, the ability of KBs to replace glucose during neuronal oxidative metabolism was fully demonstrated only recently^5^, and no evidence of direct KB oxidation by neurons to sustain memory formation has been reported yet. In mammals, the body’s main KB provider is the liver, in which acetyl-CoA used for ketogenesis is produced by β-oxidation of fatty acids (FAs) imported into the mitochondria^6^. Although there is no evidence of ketogenesis in neurons^7^, several in vitro studies in mammals have shown that astrocytes can synthesize KBs due to their ability to oxidize FAs^8,9^, suggesting that a system for local production and delivery of KBs could exist inside the brain^10^. However, it is unknown whether glia provide KBs to neurons in vivo to sustain higher brain functions.

Using *Drosophila melanogaster* and an associative olfactory memory paradigm, we investigated in vivo the metabolic communication between neurons and glia during starvation. Flies can form long-lasting olfactory aversive memories as a result of several presentations of an odorant paired with electric shocks, the negative stimuli^11^. This association is stored as a memory trace in the mushroom body (MB)^12^, the major integrative brain centre for learning and memory in insects^13^. We showed in flies fed ad libitum that the formation of protein synthesis-dependent long-term memory (LTM) after multiple spaced olfactory trainings crucially relies on the regulation of both pyruvate (a glucose derivative) metabolism in MB neurons^14^ and glucose metabolism in glial cells^15^. When flies are starved, LTM formation is blocked, which is beneficial for surviving food restriction^16^. This adaptive plasticity is specific to LTM, as starved flies maintain their ability to form consolidated—but protein synthesis-independent—memory after multiple massed trainings. Because the starved brain cannot rely on glucose as it does in the fed state, this prompted us to investigate the specific metabolic pathways at play during starvation in the MB. Our results establish that, during starvation, MB neurons import and use KBs as an energy substrate to sustain associative memory formation, a memory that we have named KB-dependent associative memory (K-AM). Additionally, we identified a local provider of KBs in the brain, the cortex glia, and show that cortex glia mobilize FAs from their own lipid droplets (LDs) to synthesize KBs. We characterized key actors in KB metabolic pathways and transport between cortex glia and MB neurons. Finally, we showed that KB production and delivery in cortex glia are regulated by AMP-activated protein kinase (AMPK), the cellular master energy sensor, thus allowing cortex glia to adapt their support to neurons depending on the brain’s energy status.

## Results

### Starved mushroom body neurons rely on ketone bodies to sustain associative memory

**Fig. 1:**
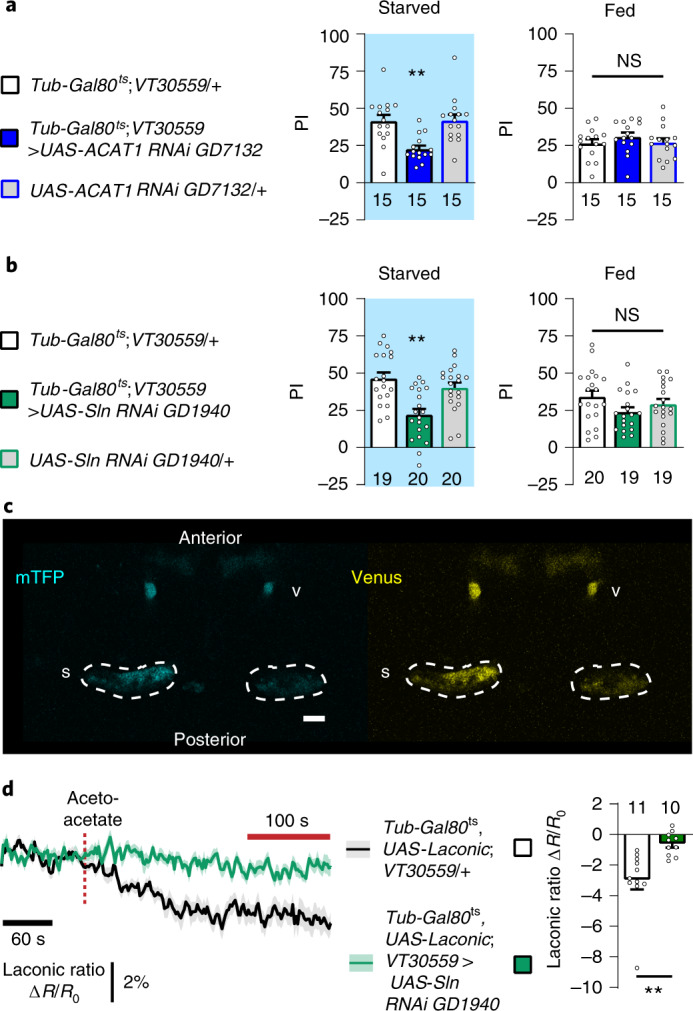
During starvation, mushroom body neurons rely on ketone body metabolism to sustain ketone body-dependent associative memory formation. **a**, Inhibition of *ACAT1* expression in adult MB neurons impaired memory after massed training in starved flies (*F*_2,42_ = 9.49, *P* = 0.0004), but not in fed flies (*F*_2,42_ = 0.74, *P* = 0.485). **b**, After massed training, memory was impaired in starved flies expressing a Sln RNAi in adult MB neurons (*F*_2,56_ = 11.33, *P* < 0.0001), but not in fed flies (*F*_2,55_ = 2.09, *P* = 0.133). **c**, Images of the Laconic FRET sensor expressed in MB neurons through the *Tub-Gal80*^*ts*^*;VT30559* driver, obtained by two-photon microscopy in the mTFP and Venus channels with the same conditions that were used for live recordings (scale bar, 20 µm). v, vertical lobe, corresponding to one of the axon fascicles of MB neurons; s, soma of MB neurons. **d**, In the fed condition, application of 10 mM of acetoacetate (red dashed line) resulted in a decreased Laconic ratio followed by a plateau in the MB soma of control flies, revealing lactate efflux from MB neuronal somas after acetoacetate bath application (mean trace ± s.e.m.). Quantification of the mean Laconic ratio at the plateau was performed on the last 100 s of recording (red line). Inhibition of *Sln* expression in adult MB neurons impaired lactate efflux evoked by acetoacetate application (*t*_19_ = 3.355, *P* = 0.003). *n* represents either a group of 40–50 flies analysed together in a behavioural assay (**a** and **b**) or the response of a single recorded fly (**d**). Data are expressed as the mean ± s.e.m. with dots as individual values, and analysed by one-way analysis of variance (ANOVA) with post hoc testing by Newman–Keuls pairwise comparisons test (**a** and **b**) or by unpaired two-sided *t*-test (**d**). Asterisks refer to the least-significant *P* value of post hoc comparison between the genotype of interest and the genotypic controls (**a** and **b**), or to the *P* value of the unpaired *t*-test comparison (**d**). **P < 0.01, NS, not significant.

**Extended Data Fig. 1:**
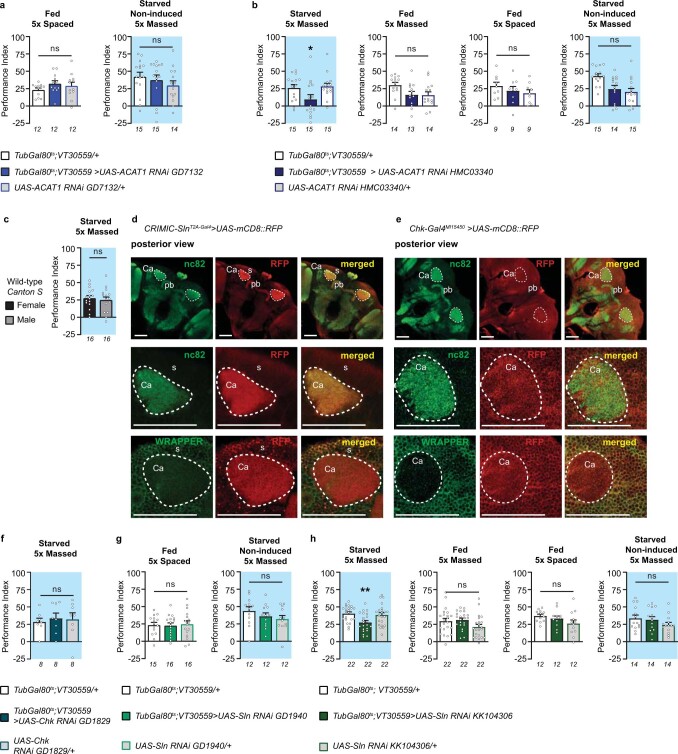
Control experiments for KB oxidation and uptake by MB neurons to form memory during starvation. (**a**) In fed flies, ACAT1 RNAi GD7132 expression did not affect memory formed after spaced training (F_2,33_ = 1.215, P = 0.310). When ACAT1 RNAi GD7132 expression was not induced, memory after massed training in starved flies was normal (F_2,41_ = 0.82, P = 0.446). (**b**) Inhibition of ACAT1 expression in adult MB neurons using a second non-overlapping RNAi impaired memory formed after massed training in starved flies (F_2,42_ = 3.54, P = 0.038), whereas ARM or LTM in fed flies was not affected (massed training: F_2,38_ = 3.38, P = 0.045, Newman-Keuls post-test is not significant; spaced training: F_2,24_ = 0.93, P = 0.407). Non-induced starved flies showed no memory defect after massed training (F_2,41_ = 7.376, P = 0.0018, Newman-Keuls post-test is not significant for comparison between *Tub-Gal80*^*ts*^*;VT30559/UAS-ACAT1 RNAi HMC03340* and the genotypic control *UAS-ACAT1 RNAi HMC03340*/+). (**c**) The memory formed after massed training in starved wild-type Canton S flies was similar between female and male flies (t_30_ = 0.45, P = 0.653). (**d**) Immunohistochemistry of *CRIMIC Sln-T2A-Gal4>UAS-mCD8::RFP* brains showing *Sln* expression pattern in red (RFP) and either pan-neuronal anti-nc82 counterstaining in green or cortex glia anti-WRAPPER counterstaining (2 top panels: nc82 and lower panel: WRAPPER). The top row displays a global view of the posterior brain (40x objective acquisition) and the lower panel shows at higher magnification (100x objective acquisition) the MB calyx region surrounded by the cortex region with MB neuronal somas. Sln is expressed in a large proportion of neurons of the central brain, and a clear expression in MB neurons can be detected in the calyx (top and middle panel: nc82 co-staining). The Sln expression pattern only partially overlaps with cortex glia labeling (lower panel: WRAPPER co-staining). pb: protocerebral bridge of Central complex, s: soma of MB neurons, Ca: Calyx of MB. (**e**) Immunohistochemistry of *Chk-Gal4*^*MI15450*^*>UAS-mCD8::GFP* brains showing the *Chk* expression pattern in red (RFP) and either pan-neuronal anti-nc82 counterstaining in green or cortex glia anti-WRAPPER counterstaining (2 top panels: nc82 and lower panel: WRAPPER). The top row displays a global view of the posterior brain (40x objective acquisition) and the lower panels show at higher magnification (100x objective acquisition) the MB calyx region surrounded by the cortex region with MB neuronal somas and cortex glia processes. Chk showed a diffuse expression in the neuropil of the central brain and a clear pattern of expression in cortex glia cells, as revealed by the honeycomb-like pattern. pb: protocerebral bridge of Central complex, s: soma of MB neurons, Ca: Calyx of MB. (**f**) Inhibition of Chk expression in adult MB neurons did not impair memory after massed training in starved flies (F_2,21_ = 0.12, P = 0.886). (**g**) In fed flies, LTM was normal when Sln expression was downregulated in adult MB neurons (F_2,44_ = 0.055, P = 0.946). When Sln RNAi GD1940 expression was not induced, memory after massed training in starved flies was normal (F_2,33_ = 1.24, P = 0.302). (**h**) Inhibition of *Sln* expression in adult MB neurons using a second non-overlapping RNAi impaired memory formed after massed training in starved flies (F_2,63_ = 5.72, P = 0.0052). By contrast, downregulation of *Sln* expression did not affect memory formation in fed flies after massed training (F_2,63_ = 2.51, P = 0.089), or after spaced training (F_2,33_ = 2.124, P = 0.13). When Sln RNAi KK104306 expression was not induced, memory formed after massed training in starved flies was normal. (F_2,39_ = 1.36, P = 0.269). n represents a group of 40–50 flies analyzed together in a behavioral assay. Data are expressed as mean ± s.e.m. with dots as individual values, and analyzed by one-way ANOVA with post hoc testing by Newman-Keuls pairwise comparisons test. Asterisks refer to the least significant P-value of *post hoc* comparison between the genotype of interest and the genotypic controls. **P<0.01, *P<0.05, ns: not significant. Scale bar: 40 µm (**d** and **e**).

To investigate the ability of MB neurons to use KBs as energy substrates for sustaining memory formation during starvation, we targeted a key enzyme of KB oxidation^17,18^, the orthologue of the human mitochondrial acetoacetyl‐CoA thiolase ACAT1, *CG10932* (DIOPT score level of homology and conservation: 15 of 15 (ref. ^19^)), using a specific RNAi. To restrict the expression of ACAT1 RNAi to adult MB, we used the *VT30559-Gal4* driver^14^ in combination with the ubiquitously expressed thermosensitive Gal4 inhibitor Gal80 (*Tub-Gal80*^*ts*^)^20^. During the 2-d induction period to allow RNAi expression in MB neurons, flies were subjected to 16 h of food deprivation just before conditioning, with a repeated massed aversive protocol to form 24-h memory under starvation^16^. After training, food deprivation was maintained until the testing of memory retrieval. Downregulation of *ACAT1* expression in the adult MB induced a strong memory impairment in starved flies (Fig. 1a). When flies were not subjected to heat activation and thus RNAi expression was not induced, memory under starvation was normal (Extended Data Fig. 1a). In fed flies, a similar repeated massed aversive protocol yielded anaesthesia-resistant memory (ARM), which is a consolidated and protein synthesis-independent memory. Contrary to what we observed in starved flies, downregulation of *ACAT1* expression in the adult MB of fed flies did not affect ARM (Fig. 1a). Finally, when a rest interval is allowed between each training cycle (spaced training), fed flies form LTM^21^. Thus, as a control, we tested that KB oxidation was not required in this other form of 24-h aversive memory in the fed condition. Downregulation of *ACAT1* expression in the adult MB did not affect LTM in fed flies (Extended Data Fig. 1a). Finally, shock reactivity and olfactory acuity controls were normal in induced starved flies (Supplementary Table 1). Using a second non-overlapping RNAi targeting *ACAT1*, we confirmed that ACAT1 is specifically required in the adult MB to form memory during starvation (Extended Data Fig. 1b and Supplementary Table 1). These data show that KB mitochondrial oxidation in MB neurons is required for memory formation during starvation, suggesting that KBs are used by neurons as an energy substrate during food deprivation periods to form consolidated memories. Because the associative memory that is formed during starvation is dependent on KB oxidation, we have named it K-AM. Because sex can influence responsiveness to diet^22^, we investigated if a sex bias could be present after K-AM formation. As the K-AM performance index (PI) was similar between male and female wild-type (Canton S) flies (Extended Data Fig. 1c) in all subsequent behavioural experiments, flies of both sexes were considered indiscriminately.

KB synthesis requires FA mitochondrial import and β-oxidation to produce the KB precursor acetyl-CoA, two steps that are considered not to occur in neurons^23^. This strongly suggests that KBs are unlikely to be synthesized in neurons^7^, but are instead imported from the extracellular space. KBs are small polar molecules that cannot freely diffuse across the plasma membrane^24^, so instead they are taken up by transporters that belong to the monocarboxylate transporter (MCT) family^24^. The *Drosophila* genome contains 22 MCT homologues that share functional properties with vertebrate MCTs^25^. Several of them are expressed in the adult brain and two of them, Silnoon (Sln) and Chaski (Chk), have been shown to efficiently transport lactate in non-neuronal cells^26,27^. However, none of them have been characterized as a KB transporter. As the mammalian KB transporters MCT2 and MCT1, expressed in neurons and glia, respectively, can transport lactate as well as KBs^28^, we decided to first investigate Sln and Chk as putative KB transporters. To determine whether these MCTs are expressed in adult MB neurons, we used a gene trap Gal4 line specific to each gene, in which the splicing acceptor T2A and the Gal4 sequence are inserted in-frame into either the first coding intron of Sln or the first and 5′ untranslated region intron of Chk (*Sln-Gal4*^*CRIMIC00519-TG4.0*^ or *Chk-Gal4*^*MI15450*^, respectively)^26,29^. The Gal4 expression pattern, which is expected to reflect the endogenous transcription patterns of the gene, was assessed with a UAS-mCD8-RFP reporter. Our results show that both Sln and Chk are expressed in neurons and cortex glia, as a red fluorescent protein signal could be detected in both the neuropil and the cortical region of the central brain (Extended Data Fig. 1d,e for Sln and Chk). While Sln was strongly expressed in MB neurons with clear labelling of the MB neuropil (nc82 co-staining panel in Extended Data Fig. 1d) and only partially overlapped with cortex glia labelling (WRAPPER co-staining panel in Extended Data Fig. 1d), Chk expression was clearly detected in cortex glia cells (WRAPPER co-staining panel in Extended Data Fig. 1e) and presented a more diffuse staining in the entire brain neuropil (nc82 co-staining panel in Extended Data Fig. 1e). Thus, to investigate if Sln or Chk could mediate KB uptake by neurons during starvation to sustain K-AM, we downregulated their expression in adult MB neurons using specific RNAi and tested the resulting memory performances. Downregulation of *Sln* in adult MB neurons induced a strong K-AM defect (Fig. 1b), whereas downregulation of *Chk* had no effect on K-AM (Extended Data Fig. 1f). In fed flies, downregulation of *Sln* in adult MB neurons had no effect on memory after massed training (Fig. 1b) or after spaced training (Extended Data Fig. 1g), showing that *Sln* is not required in the adult MB for consolidated types of aversive memory in fed flies. In the absence of RNAi induction, flies showed normal K-AM (Extended Data Fig. 1g). Furthermore, sensory controls were normal in induced starved flies (Supplementary Table 1). Similar results were obtained using a second non-overlapping RNAi targeting *Sln* (Extended Data Fig. 1h and Supplementary Table 1), confirming that Sln in the adult MB is specifically required for K-AM formation. These data suggest that Sln may be the MCT that transports KBs into MB neurons during starvation.

**Extended Data Fig. 2:**
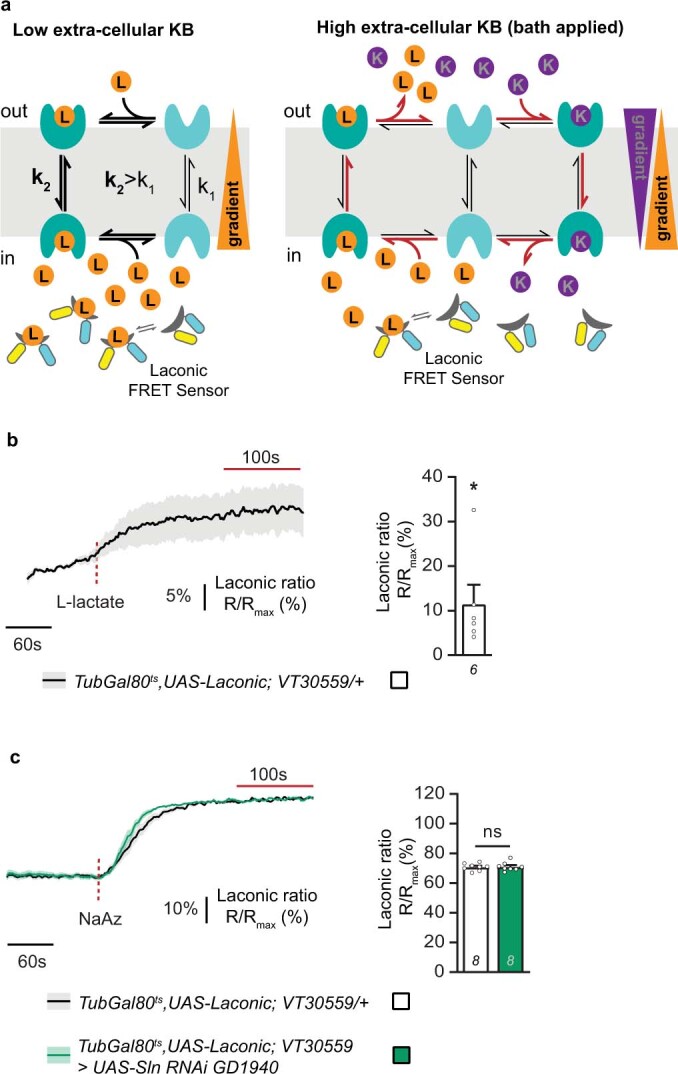
Control experiments for KB uptake by MB neurons. (**a**) MCT switches to the other side of the membrane at a higher rate when a substrate is bound (MCT – L; k2) than when no substrate is bound (MCT; k1), with k1<k2. Trans-acceleration occurs because the conformational switch of MCT across the cell membrane is facilitated when an adequate substrate such as KB is bound. Lactate efflux can be monitored indirectly by an intracellular Laconic FRET sensor that is sensitive to lactate concentration. When KB is applied, the high concentration of KB in the extracellular media increases the rate of lactate transport in the opposite direction, corresponding here to lactate efflux. (**b**) In fed flies expressing the intracellular Laconic FRET sensor in MB neurons, application of 10 mM of L-lactate on the brain induced a strong increase in the Laconic FRET ratio showing that the intracellular lactate concentration subsequently increased in response to lactate uptake by MB neurons (t_5_ = 2.627, P = 0.047). Thus, as previously shown by several other research groups, the Laconic FRET sensor can be used to monitor the intracellular lactate level. (**c**) To assess that *Sln* downregulation in MB neurons do not affect the lactate intracellular concentration to a level below the Laconic FRET sensor sensitivity threshold, thus preventing detection of any Laconic FRET ratio change in Fig. 2d, we applied a saturation treatment to reach a maximal plateau of Laconic FRET ratio (that is saturation of the sensor) that was used to normalize the different genotypic conditions. In fed flies expressing the Laconic FRET sensor in MB neurons, application of 5 mM NaAz, a strong inhibitor of mitochondrial respiration, induced a strong increase in the Laconic FRET ratio, reaching the saturation level of the sensor (mean trace ± s.e.m.). The last 100 s of recording (red bar), when the Laconic FRET sensor had reached saturation, were used to normalize; the initial Laconic ratio was measured during the 120-s baseline recording before NaAz application. In fed flies, the lactate basal concentration in MB neurons of flies expressing *Sln* RNAi was similar to the genotypic control (t_14_ = 0.2091, p = 0.837). n represents the response of a single recorded fly. Data are expressed as mean ± s.e.m. with dots as individual values, and analyzed by one-sample two-sided t-test with theoretical mean = 0 (**b**) or unpaired two sided two-sample t-test (**c**). Asterisks refer to the P-value of the t-test comparison. *P<0.05, ns: not significant.

To assess the ability of Sln to transport KBs in vivo, we used the trans-acceleration property of MCT—also known as accelerated-exchange transport—in which the presence of extracellular monocarboxylates stimulates transporter substrate efflux^30^. In our experimental conditions, the extracellular monocarboxylate was the KB acetoacetate and the intracellular monocarboxylate was lactate, which we monitored using the Laconic FRET sensor^22,31^ (Extended Data Fig. 2a,b). Thus, in this experimental condition, the artificially elevated level of extracellular acetoacetate should lead to an efflux of lactate through the appropriate MCT. In the normal condition, this effect is monitored by a decrease in intracellular lactate concentration (detected as a decrease in the Laconic FRET ratio), whereas when the expression of MCT transporting acetoacetate is downregulated this lactate efflux should be abolished (detected as no change in the Laconic FRET ratio), or at least decreased. When acetoacetate was bath-applied on fly brains expressing the Laconic FRET sensor in adult MB neurons (Fig. 1c), the Laconic ratio decreased in MB neuronal somas (Fig. 1d), suggesting that an efflux of lactate towards the extracellular medium occurred as predicted by the trans-acceleration model. When acetoacetate was applied on flies expressing Sln RNAi in the adult MB, the decrease in Laconic ratio was almost abolished (Fig. 1d), suggesting that the trans-acceleration of lactate efflux by acetoacetate requires Sln in MB neurons. As the lactate basal concentration of MB neurons was not changed by Sln downregulation (Extended Data Fig. 2c), these imaging experiments show that Sln can function as a KB transporter in MB neurons (Fig. 1d). Combined with the behavioural experiments (Fig. 1b), these imaging data strongly support the view that, during starvation, Sln is the MCT required for KB import into MB neurons to sustain K-AM formation.

### In starved flies, ketone bodies provided to mushroom body neurons originate from cortex glia

**Fig. 2:**
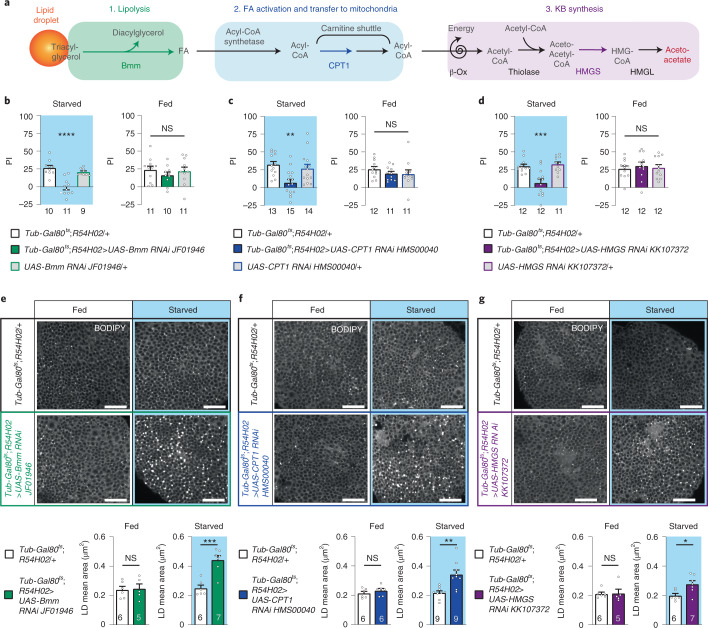
During starvation, cortex glia mobilize their own fatty acid store to provide ketone bodies to sustain ketone body-dependent associative memory. **a**, KB production pathway. Triacylglycerols stored in LDs are hydrolysed by the lipase Bmm into FAs and diacylglycerol. FAs are then activated by an acyl-CoA synthetase and imported as acyl-CoA into the mitochondria by the carnitine shuttle system whose CPT1 is a component. Then acyl-CoA enters the β-oxidation cycle to produce acetyl-CoA that will be used to generate acetoacetate by the successive actions of a thiolase, the HMGS and the HMG-CoA lyase. **b**–**d**, Downregulation in adult cortex glia of each of the three key enzymes of KB production, Bmm (**b**), CPT1 (**c**) and HMGS (**d**) impaired K-AM (Bmm: *F*_2,27_ = 23.29, *P* < 0.0001; CPT1: *F*_2,39_ = 7.304, *P* = 0.002; HMGS: *F*_2,32_ = 12.66, *P* < 0.0001) but not ARM in fed flies (Bmm: *F*_2,29_ = 0.58, *P* = 0.567; CPT1: *F*_2,31_ = 0.825, *P* = 0.448, HMGS: *F*_2,33_ = 0.21, *P* = 0.815). **e**–**g**, BODIPY LD staining and quantification in starved and fed flies expressing or not an RNAi targeting one of the three key enzymes of KB production in adult cortex glia. **e**, Inhibition of *Bmm* expression in adult cortex glia of fed flies did not change the mean area of LDs observed in the brain region where cortex glia enwrap MB neuronal soma (*t*_9_ = 0.193, *P* = 0.851), whereas larger LDs were observed in starved *Bmm*^*RNAi*^ expressing flies compared to controls (*t*_11_ = 5.085, *P* = 0.0004). **f**,**g**, Similarly, inhibition of either *CPT1* or *HMGS* expression in adult cortex glia had no effect on LD mean area in the fed condition (CPT1: *t*_10_ = 0.950, *P* = 0.364; HMGS: *t*_9_ = 0.121, *P* = 0.907), whereas during starvation an increase in LD mean area was observed as compared to control flies (CPT1: *t*_16_ = 3.792, *P* = 0.0016 ; HMGS: *t*_11_ = 2.690, *P* = 0.021). *n* represents either a group of 40–50 flies analysed together in a behavioural assay (**b**–**d**) or one BODIPY-stained brain (**e**–**g**). Data are expressed as the mean ± s.e.m. with dots as individual values, and analysed by one-way ANOVA with post hoc testing by Newman–Keuls pairwise comparisons test (**b**–**d**) or by unpaired two-sided *t*-test (**e**–**g**). Asterisks refer to the least-significant *P* value of a post hoc comparison between the genotype of interest and the genotypic controls or to the *P* value of the unpaired *t*-test comparison. ****P < 0.0001, ***P < 0.001, **P < 0.01, *P < 0.05. Scale bar, 20 µm.

**Extended Data Fig. 3:**
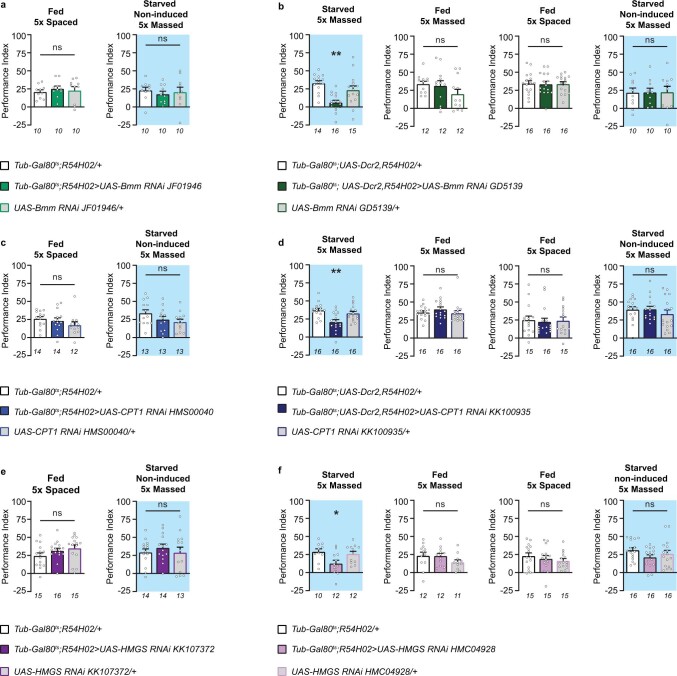
Control experiments for KB production by cortex glia to sustain K-AM formation in MB neurons. (**a**) In fed flies when Bmm expression was downregulated in cortex glia LTM was normal (F_2,27_ = 0.29, P = 0.749). When RNAi expression was not induced, K-AM was normal (F_2,27_ = 0.27, P = 0.768). (**b**) A second non-overlapping RNAi targeting *Bmm* (Bmm RNAi GD5139) was used to confirm the specific K-AM defect. In order to increase RNAi efficiency, Dicer2 expression was induced together with the RNAi expression in adult cortex glia using the *Tub-Gal80*^*ts*^*; UAS-Dcr2, R54H02* line. Inhibition of *Bmm* expression in adult cortex glia using Bmm RNAi GD5139 impaired K-AM in starved flies (F_2,42_ = 10.70, P = 0.0002), whereas ARM or LTM in fed flies was not affected (ARM: F_2,33_ = 1.55, P = 0.228; LTM: F_2,45_ = 0.02, P = 0.982). Non-induced starved flies showed no K-AM defect (F_2,27_ = 0.003, P = 0.997). (**c**) Downregulation of CPT1 expression in adult cortex glia did not affect LTM (F_2,37_ = 1.32, P = 0.279). When RNAi expression was not induced, K-AM was normal (F_2,36_ = 2.12, P = 0.135). (**d**) A second non-overlapping RNAi targeting *CPT1* (CPT1 RNAi KK100935) was used to confirm the specific K-AM defect. As for Bmm RNAi GD5139, we used the *Tub-Gal80*^*ts*^*; UAS-Dcr2, R54H02* line to increase RNAi efficiency. Inhibition of *CPT1* expression in adult cortex glia using CPT1 RNAi KK100935 impaired K-AM (F_2,45_ = 8.72, P = 0.0006), whereas ARM or LTM in fed flies was not affected (ARM: F_2,45_ = 0.87, P = 0.424; LTM: F_2,43_ = 0.06, P = 0.939). Non-induced starved flies showed no K-AM defect (F_2,45_ = 0.66, P = 0.524). (**e**) When *HMGS* expression is downregulated in the cortex, LTM was normal (F_2,43_ = 1.74, P = 0.187). When RNAi expression was not induced, K-AM was normal (F_2,38_ = 0.43, P = 0.656). (**f**) A second non-overlapping RNAi targeting *HMGS* (HMGS RNAi HMC04928) was used to confirm the specific K-AM defect. Inhibition of *HMGS* expression in adult cortex glia using this RNAi impaired K-AM (F_2,31_ = 4.53, P = 0.019), whereas ARM or LTM in fed flies was not affected (ARM: F_2,32_ = 1.56, P = 0.227; LTM: F_2,42_ = 0.72, P = 0.494). Non-induced starved flies showed no K-AM defect (F_2,45_ = 1.58, P = 0.217). n represents a group of 40–50 flies analyzed together in a behavioral assay. Data are expressed as mean ± s.e.m. with dots as individual values, and analyzed by one-way ANOVA with post hoc testing by Newman-Keuls pairwise comparisons test. Asterisks refer to the least significant P-value of *post hoc* comparison between the genotype of interest and the genotypic controls. **P<0.01, *P<0.05, ns: not significant.

It has been proposed that *Drosophila* glia synthesize KBs from their own lipid stores^32^. These internal lipid stores, known as LDs^33^, are mainly composed of triacylglycerol, phospholipids and sterols^34^. Among the different glial cell types present in the *Drosophila* brain, two types in the larval stage contain an abundance of LDs^35^: the surface glia, which are functionally equivalent to the blood–brain barrier; and the cortex glia, which are in direct contact with neurons. Indeed, approximately 2,600 cortex glia cells individually insulate each neuronal soma in a honeycomb-like network of glial processes, with each cortex glial cell enwrapping up to 100 neuronal somas^36,37^. Thus, we investigated if cortex glia could be the cells responsible for providing KBs to neurons to sustain K-AM during starvation. KB production from LDs can be divided into three sequential steps: (1) lipolysis, to mobilize FAs from LDs; (2) FA import into glia mitochondria; and (3) mitochondrial acetyl-CoA production and ketogenesis^34,38^ (Fig. 2a). To test our hypothesis, we targeted a key enzyme from each of these three steps in adult cortex glia using specific RNAi under the control of an inducible cortex glia driver^15,36^ (*R54H02-Gal4* in combination with *Tub-Gal80*^*ts*^) and then examined the K-AM of starved flies (Fig. 2a–d). The lipolysis of LD triacylglycerol into glycerol and FAs occurs through the sequential action of three FA lipases. Because Brummer (Bmm), the orthologue of the mammalian adipose triglyceride lipase (ATGL), catalyses the first lipolysis reaction^39^, we selected it to test our hypothesis. During starvation, downregulation of *Bmm* expression in adult cortex glia resulted in a strong K-AM impairment, whereas memory after massed training in fed flies was normal (Fig. 2b). Additionally, K-AM was normal in the absence of RNAi induction, and Bmm was not required in cortex glia for LTM (Extended Data Fig. 3a; see Supplementary Table 2 for sensory controls and also Extended Data Fig. 3b obtained with a second non-overlapping RNAi targeting *Bmm*), which shows that Bmm in the adult cortex glia is specifically required for K-AM formation.

In a second step, FAs need to be imported into mitochondria for subsequent β-oxidation and acetyl-CoA production^17^. As carnitine palmitoyltransferase 1 (CPT1), the outer mitochondrial membrane component of the FA transport system^40^, catalyses the rate-limiting step of FA import for KB synthesis^10^, we tested its involvement in cortex glia for K-AM. During starvation, downregulation of *CPT1* expression in adult cortex glia resulted in strong K-AM impairment, whereas memory after massed training in fed flies was normal (Fig. 2c). Additionally, K-AM was normal in the absence of RNAi induction, and CPT1 was not required in cortex glia for LTM (Extended Data Fig. 3c; see Supplementary Table 2 for sensory controls and also Extended Data Fig. 3d obtained with a second non-overlapping RNAi targeting *CPT1*), which shows that CPT1 in the adult cortex glia is specifically required for K-AM formation.

Once inside the mitochondrial matrix, activated FAs enter the β-oxidation cycle to produce acetyl-CoA. Acetyl-CoA is then used to generate acetoacetate, which can be further reduced to β-hydroxybutyrate^38^. As the rate-limiting step of this ketogenesis pathway is catalysed by HMG-CoA synthase (HMGS)^38^, we tested its requirement in cortex glia for K-AM. During starvation, downregulation of *HMGS* expression in adult cortex glia resulted in a strong K-AM impairment, whereas memory after massed training in fed flies was normal (Fig. 2d). Additionally, K-AM was normal in the absence of RNAi induction, and HMGS was not required in cortex glia for LTM (Extended Data Fig. 3e; see Supplementary Table 2 for sensory controls and also Extended Data Fig. 3f obtained with a second non-overlapping RNAi targeting *HMGS*), which shows that HMGS in the adult cortex glia is specifically required for K-AM formation.

**Extended Data Fig. 4:**
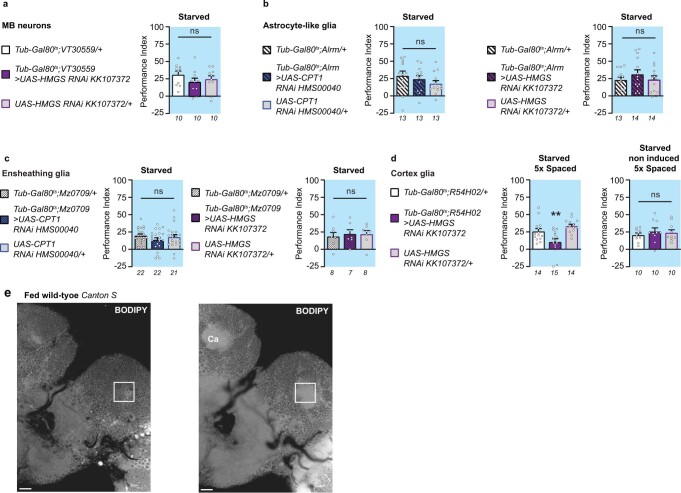
KB production is not required in other glial cells type or MB neurons to sustain K-AM. (**a**) When *HMGS* expression was downregulated in adult MB neurons, K-AM was normal (F_2,27_ = 1.068, P = 0.358). (**b**) When either *CPT1* or *HMGS* expression was downregulated in adult astrocyte-like glia, K-AM was normal (CPT1: F_2,36_ = 1.166, P = 0.323; HMGS: F_2,38_ = 0.778, P = 0.466). (**c**) When either *CPT1* or *HMGS* expression was downregulated in adult ensheathing glia, K-AM was normal (CPT1: nF_2,62_ = 1.189, P = 0.311; HMGS: F_2,20_ = 0.139, P = 0.871). (**d**) In starved flies, memory formed after spaced training is impaired when *HMGS* is downregulated in cortex glia (F_2,40_ = 9.46, P = 0.0004). When RNAi expression was not induced, memory formed after spaced training in starved flies was normal (F_2,27_ = 0.39, P = 0.678). (**e**) Full view of a fed wild-type Canton S fly central brain stained with BODIPY, as in Fig. 2e-g. The left panel shows a confocal plane in which the ROI (white square) for subsequent quantification of LDs has been selected. The right panel shows a confocal plane from the same brain but 4 µm deeper, showing the ROI position toward the MB calyx that is used as a landmark to define the ROI x-y axis position. n represents a group of 40–50 flies analyzed together in a behavioral assay. Data are expressed as mean ± s.e.m. with dots as individual values, and analyzed by one-way ANOVA with post hoc testing by Newman-Keuls pairwise comparisons test. Asterisks refer to the least significant P-value of *post hoc* comparison between the genotype of interest and the genotypic controls. **P<0.01, ns: not significant. Scale bar: 40 µm (**e**).

Altogether, these data show that, during starvation, the three steps of KB production (LD lipolysis, FA mitochondrial import and ketogenesis) are required in cortex glia to support K-AM formation in MB neurons. Because LDs have been described in other cells types^41,42^, we downregulated KB production pathways in either MB neurons or other types of glial cells and found that K-AM was normal (Extended Data Fig. 4a-c). These results support the hypothesis that cortex glia are the main provider of KBs to neurons during starvation to sustain K-AM.

In the fed condition, massed and spaced training elicit different types of memory relying on different metabolic pathways^14^. In flies subjected to starvation, downregulation of KB production in cortex glia resulted in a specific 24-h memory defect after either massed (Fig. 2b–d) or spaced (Extended Data Fig. 4d) training. Thus, independent of the training protocol, the formation of persistent memory in starved flies relies on KB production by cortex glia.

Because LDs are an internal reservoir of KB precursors, we aimed to confirm the hypothesis that the cortex glia are a local provider of KBs to neurons, by assessing the LD content of fly brains in which KB production has been specifically downregulated in cortex glia. We used the BODIPY 493/503 probe to stain LDs, as previously used in the *Drosophila* visual system^43^. LDs of approximately 0.2 µm² in area could be observed in fed flies, in the brain region of neuronal somas where they are enwrapped by cortex glial processes (Fig. 2e–g and Extended Data Fig. 4e). In fed flies, downregulation of the KB production pathway genes *Bmm*, *CPT1* and *HMGS* in cortex glia did not change the mean area of LDs as compared to the genotypic controls, suggesting that LDs are not used to synthesize KBs in fed flies. On the contrary, in the starved condition, the downregulation of *Bmm*, *CPT1* or *HMGS* significantly increased the mean area of LDs compared to the genotypic controls (Fig. 2e–g for *Bmm*, *CPT1* or *HMGS*). Importantly, in genotypic controls, starvation did not induce any increase in cortex glia LD content, suggesting that FA uptake and storage in LDs and their consumption are at steady state. Altogether, these data suggest that when KB production is downregulated in cortex glia during starvation, FAs from LDs are not efficiently mobilized; that is, instead of being used to provide energy and produce KBs, they just accumulate. Thus, mobilization of cortex glia FA internal stores during starvation and their use for ketogenesis constitute an entire metabolic pathway that is required to sustain K-AM formation in neurons, strongly suggesting that cortex glia are the main local provider of KBs to support neuronal function during starvation.

### Ketone bodies are exported from cortex glia via Chk to sustain ketone body-dependent associative memory

**Fig. 3:**
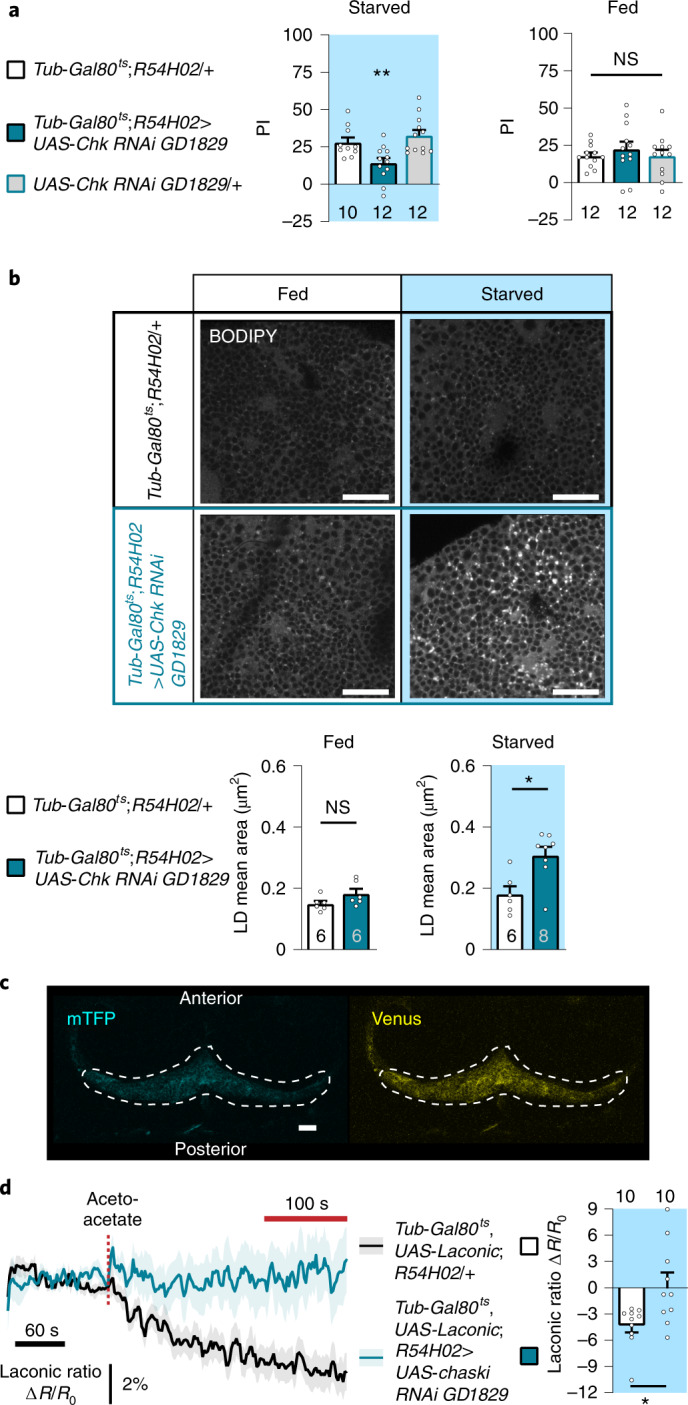
Chaski is the cortex glia monocarboxylate transporter required to provide ketone bodies to sustain ketone body-dependent associative memory. **a**, Inhibition of *Chk* expression in adult cortex glia impaired starved K-AM (*F*_2,31_ = 8.22, *P* = 0.001), while memory after massed training in fed flies was normal (*F*_2,33_ = 0.43, *P* = 0.653). **b**, BODIPY LD staining and quantification in starved and fed flies expressing or not the Chk RNAi in adult cortex glia. Inhibition of *Chk* expression in adult cortex glia in fed flies did not change LD mean area (*t*_10_ = 1.785, *P* = 0.105), whereas during starvation an increase in LD mean area was observed as compared to controls (*t*_12_ = 3.181, *P* = 0.008). **c**, Images of the Laconic sensor expressed in cortex glia with the *Tub-Gal80*^*ts*^*;R54H02* driver, obtained by two-photon microscopy in the mTFP and Venus channels, under the same conditions as those used for live recordings. **d**, Application of 10 mM acetoacetate (red dashed line) resulted in a decreased Laconic ratio followed by a plateau in cortex glia of starved control flies, showing lactate efflux from cortex glia after acetoacetate bath application (mean trace ± s.e.m. of ten recordings per genotype). Quantification of the mean Laconic ratio at the plateau was performed during the last 100 s of recording (red line). Inhibition of *Chk* expression in adult cortex glia impaired this lactate efflux evoked by acetoacetate application (*t*_18_ = 2.791, *P* = 0.012). *n* indicated within the graph represents a group of 40–50 flies analysed together in a behavioural assay (**a**), one BODIPY-stained brain (**b**) or the response of a single recorded fly (**d**). Data are expressed as the mean ± s.e.m. with dots as individual values, and analysed by one-way ANOVA with post hoc testing by Newman–Keuls pairwise comparisons test (**a**) or by unpaired two-sided *t*-test (**b** and **d**). Asterisks refer to the least-significant *P* value of a post hoc comparison between the genotype of interest and the genotypic controls (**a**) or to the *P* value of the unpaired *t*-test comparison (**b** and **d**). **P < 0.001, *P < 0.05. Scale bar, 20 µm (**b** and **c**).

**Extended Data Fig. 5:**
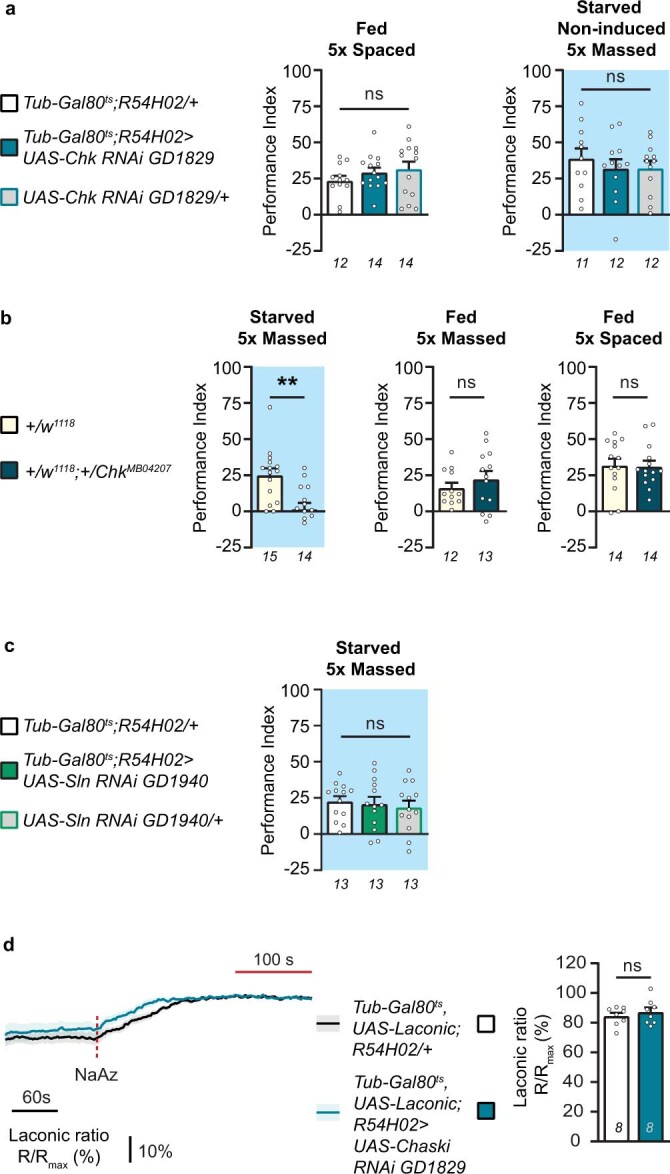
Control experiments for KB export by cortex glia to sustain K-AM formation in MB neurons. (**a**) In fed flies, when Chk expression was downregulated in adult cortex glia, LTM was normal (F_2,37_ = 0.96, P = 0.393). When Chk RNAi expression was not induced, K-AM was normal (F_2,32_ = 0.40, P = 0.677). (**b**) Since starvation induced strong lethality in *Chk*^*MB04207*^ homozygous flies, we used heterozygous *Chk*^*MB04207*^/+ flies to confirm Chk function in K-AM. Furthermore, since both *Chk*^*MB04207*^ flies (which are in a *w*^*1118*^ background) and *w*^*1118*^ flies have lower memory scores, we only counted heterozygous females (*w*^*1118*^*/+*; *Chk*^*MB04207*^*/+*) and not hemizygous males (*w*^*1118*^*/Y*; *Chk*^*MB04207*^*/+*), and counted only heterozygous *w*^*1118*^/+ females as control flies. Heterozygous *w*^*1118*^*/+*; *Chk*^*MB04207*^*/+* female flies had a strong K-AM defect as compared to control *w*^*1118*^*/+;+* female flies (t_27_ = 3.24, P = 0.0032). Neither ARM (t_23_ = 0.89, P = 0.379) nor LTM (t_26_ = 0.10, P = 0.918) was affected in these heterozygous *w*^*1118*^/+;*Chk*^*MB04207*^/+ female flies. (**c**) Inhibition of Sln expression in adult cortex glia did not impair K-AM (F_2,36_ = 0.23, P = 0.794). (**d**) In starved flies expressing the Laconic FRET sensor in cortex glia, application of 5 mM NaAz induced a strong increase in the Laconic FRET ratio, reaching the saturation level of the sensor (mean trace ± s.e.m.). The last 100 s of recording (red bar), when the Laconic FRET sensor had reached saturation, were used to normalize; the initial Laconic ratio was measured during the 2-min baseline recording before NaAz application. In starved flies, the lactate basal concentration in cortex glia expressing Chk RNAi was similar to the genotypic control (t_14_ = 0.743, P = 0.469). n represents either a group of 40–50 flies analyzed together in a behavioral assay (**a**–**c**) or the response of a single recorded fly (**d**). Data are expressed as mean ± s.e.m. with dots as individual values, and analyzed by one-way ANOVA with post hoc testing by Newman-Keuls pairwise comparisons test (**a** and **c**) or by unpaired two-sided t-test (**b** and **d**). Asterisks refer to the least significant P-value of *post hoc* comparison between the genotype of interest and the genotypic controls (**a** and **c**) or to the P-value of the unpaired t-test comparison (**b** and **d**). **P<0.01, ns: not significant.

As demonstrated for the neuronal import of KBs via Sln, KB export from cortex glia needs to be mediated by an MCT. During starvation, the decreased survival observed in *Chk* mutants is rescued by specific expression of *Chk* in glial cells^26^, suggesting that this MCT is functionally relevant in glial cells during the starvation state. Moreover, the expression pattern of *Chk* presented previously shows overlap with the cortex glial marker WRAPPER, strongly suggesting that Chk is not only expressed in MB neurons but also in adult cortex glia (Extended Data Fig. 1e). We therefore tested if Chk is the MCT required for KB export to sustain K-AM in MB neurons during starvation. Downregulation of *Chk* expression in adult cortex glia resulted in a strong K-AM defect, whereas after massed training, fed flies had normal ARM (Fig. 3a). In the absence of induction, K-AM was normal and Chk was not required in cortex glia for LTM (Extended Data Fig. 5a). In induced starved flies, sensory controls were normal (Supplementary Table 3). To confirm that Chk is required in cortex glia to sustain K-AM, and because no other RNAi targeting Chk was available, we used the *Chk*^*MB04207*^ constitutive mutant previously used by Delgado et al.^26^. However, because starvation induced strong lethality in homozygous *Chk*^*MB04207*^ flies^26^, we investigated memories in heterozygous *Chk*^*MB04207*^/+ flies. Heterozygous *Chk*^*MB04207*^/+ flies displayed a strong K-AM defect in comparison to control flies, whereas ARM and LTM were normal as well as the sensory controls (Extended Data Fig. 5b and Supplementary Table 3), thus confirming that Chk is required to sustain K-AM. To assess that the Chk function in KB export to support K-AM was specific to this MCT, we tested the effect of *Sln* downregulation in cortex glia on K-AM. Downregulation of *Sln* in cortex glia did not affect K-AM (Extended Data Fig. 5c). To reinforce our results pointing to Chk as the KB transporter in cortex glia during starvation, we assessed the LD content of fly brains in which *Chk* expression is downregulated in cortex glia, as we did for genes involved in KB production (Fig. 2e–g). We hypothesized that if the output of the metabolic pathway of KB production is blocked, then FA should accumulate in the LDs of these cells during starvation. In the fed state, no difference in LD content was observed between flies expressing Chk RNAi or not in cortex glia (Fig. 3b). However, in starved flies, the downregulation of *Chk* in adult cortex glia resulted in an increase in the mean area of LDs compared to the genotypic control (Fig. 3b). These data show that when KB export is downregulated in cortex glia during starvation, FAs from LDs are not efficiently mobilized and accumulate, suggesting a negative feedback effect on the entire KB production pathway.

To assess the ability of Chk to transport KBs, we used the same strategy as with Sln, based on the trans-acceleration property of MCTs (Extended Data Fig. 2a). The Laconic FRET sensor in cortex glia (Fig. 3c) was expressed to monitor the efflux of lactate from these cells during bath application of acetoacetate. As before, if Chk is able to transport KB, then extracellular acetoacetate as a substrate of MCT should increase the efflux of lactate, whereas this lactate efflux should be abolished when Chk is not present. When acetoacetate was bath-applied on the brains of starved flies, a decrease in the Laconic ratio was observed in cortex glia (Fig. 3d), suggesting that an efflux of lactate towards the extracellular medium occurred as predicted by the trans-acceleration model. When acetoacetate was applied on starved flies expressing Chk RNAi in the adult cortex glia, this decrease in Laconic ratio was abolished (Fig. 3d), and as Chk downregulation in cortex glia did not affect lactate basal concentration in those cells (Extended Data Fig. 5d), we conclude that the trans-acceleration of lactate efflux by acetoacetate requires Chk in cortex glia. Altogether, these data show that Chk is required in cortex glia to export KBs, the final product of FA mobilization, which is critical for K-AM during starvation.

### During starvation, AMP-activated protein kinase regulates cortex glia ketone body supply to mushroom body neurons

**Fig. 4:**
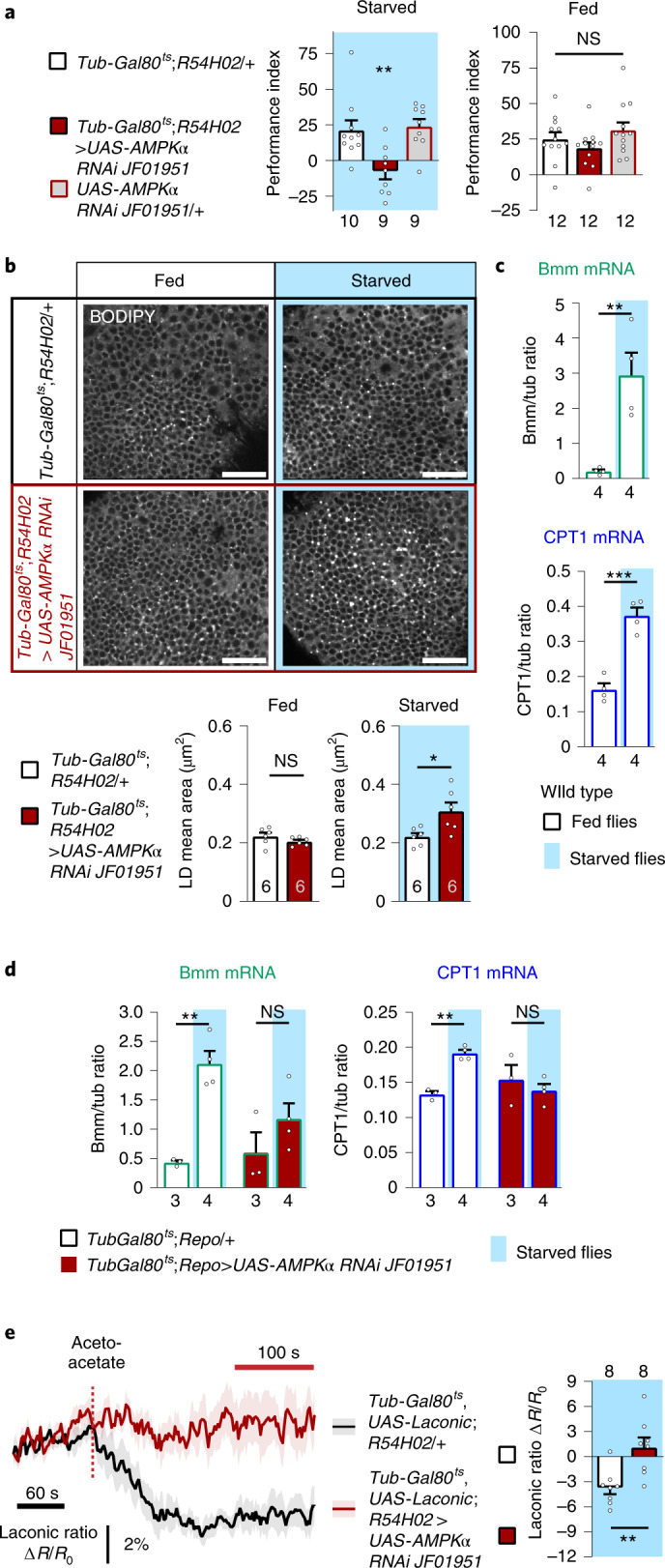
During starvation, AMPK is required in cortex glia for ketone body production and export to sustain ketone body-dependent associative memory in neurons. **a**, Inhibition of *AMPKα* expression in adult cortex glia impaired K-AM (*F*_2,25_ = 8.05, *P* = 0.002), while ARM was normal in fed flies (*F*_2,33_ = 1.76, *P* = 0.189). **b**, BODIPY LD staining and quantification in starved and fed flies expressing or not an AMPK*α* RNAi in cortex glia. In fed flies, inhibition of *AMPKα* expression in adult cortex glia did not change the LD mean area (*t*_10_ = 1.308, *P* = 0.220), whereas an increase in LD mean area was observed in starved flies as compared to controls (*t*_10_ = 2.660, *P* = 0.0239). **c**, Starvation strongly increased Bmm and CPT1 mRNA levels (Bmm: *t*_6_ = 4.25, *P* = 0.0054; CPT1: *t*_6_ = 7.28, *P* = 0.0003). **d**, In starved flies expressing AMPK RNAi in glial cells, Bmm and CPT1 mRNA levels did not differ from those of fed flies (Bmm: *t*_5_ = 1.34, *P* = 0.238; CPT1: *t*_5_ = 0.76, *P* = 0.482), whereas in the genotypic control groups, starvation induced a significant increase in each gene’s mRNA level (Bmm: *t*_5_ = 6.54, *P* = 0.001; CPT1: *t*_5_ = 8.55, *P* = 0.0004). **e**, Application of 10 mM acetoacetate (red dashed line) resulted in a decreased Laconic ratio followed by a plateau in cortex glia of starved control flies, showing lactate efflux from cortex glia after acetoacetate bath application (mean trace ± s.e.m.). Quantification of the mean Laconic ratio at the plateau was performed during the last 100 s of recording (red line). Inhibition of *AMPKα* expression in adult cortex glia impaired this lactate efflux evoked by acetoacetate application (*t*_14_ = 3.393, *P* = 0.004). *n* represents a group of 40–50 flies analysed together in a behavioural assay (**a**), one BODIPY-stained brain (**b**), mRNA extracted from a group of 50 flies (**c** and **d**) or the response of a single recorded fly (**e**). Data are expressed as the mean ± s.e.m. with dots as individual values, and analysed by one-way ANOVA with post hoc testing by Newman–Keuls pairwise comparisons test (**a**) or by unpaired two-sided *t*-test (**b**–**e**). Asterisks refer to the least-significant *P* value of a post hoc comparison between the genotype of interest and the genotypic controls (**a**) or the *P* value of the unpaired *t*-test comparison (**b**–**e**). ***P < 0.001, **P < 0.01, *P < 0.05. Scale bar, 20 µm (**b**).

**Extended Data Fig. 6:**
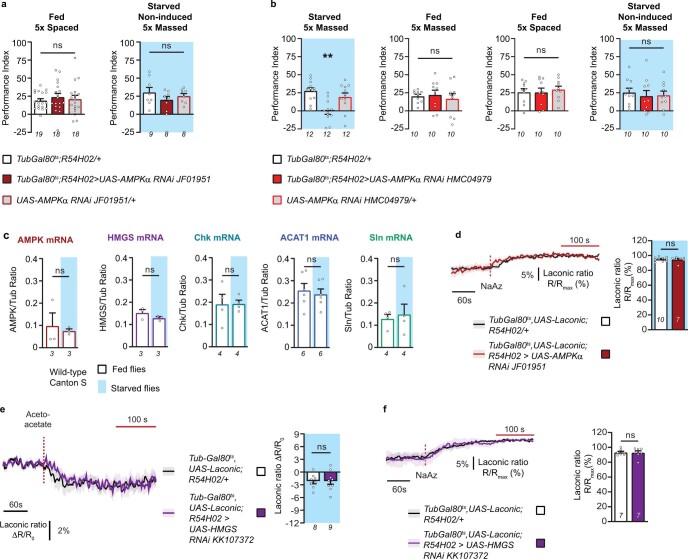
Control experiments for AMPK requirement in cortex glia to sustain K-AM formation in MB neurons. (**a**) In fed flies, *AMPKα* expression in cortex glia is not required for LTM (F_2,52_ = 0.33, P = 0.717). When AMPK*α* RNAi expression was not induced, K-AM was normal (F_2,21_ = 1.02, P = 0.378). (**b**) Inhibition of *AMPKα* expression in adult cortex glia using a second non-overlapping RNAi impaired K-AM in starved flies (F_2,33_ = 9.95, P = 0.0004), whereas memory formed in fed flies after either massed or spaced training was not affected (ARM: F_2,27_ = 0.23, P = 0.799; LTM: F_2,27_ = 0.187, P = 0.831). Non-induced flies showed no K-AM defect (F_2,27_ = 0.16, P = 0.855). (**c**) In wild-type Canton S fly heads, starvation did not change the mRNA level of either AMPK, HMGS, Chk, ACAT1 or Sln (AMPK: t_4_ = 0.382, p = 0.722, HMGS: t_4_ = 1.40, p = 0.24; Chk t_6_ = 0.033, p = 0.98; ACAT1 t_10_ = 0.439, p = 0.669; Sln: t_6_ = 0.389, p = 0.711). (**d**) In starved flies expressing the Laconic FRET sensor in cortex glia, application of 5 mM NaAz induced a strong increase in the Laconic FRET ratio, reaching the saturation level of the sensor (mean trace ± s.e.m.). The last 100 s of recording (red bar), when the Laconic FRET sensor had reached saturation, were used to normalize; the initial Laconic ratio was measured during the 2-min baseline recording before NaAz application. In starved flies, the lactate basal concentration in cortex glia expressing AMPK*α* RNAi was similar to the genotypic control (AMPK: t_15_ = 0.279, P = 0.784). (**e**) Application of 10 mM acetoacetate (red dashed line) results in a decreased Laconic ratio followed by a plateau in cortex glia of starved control flies, showing lactate efflux from cortex glia after acetoacetate bath application (mean trace ± s.e.m.). Inhibition of *HMGS* expression did not change the lactate efflux evoked by acetoacetate application (t_15_ = 0.004, P = 0.99). Quantification of the mean Laconic ratio at the plateau was performed during the last 100 s of recording (red line). (**f**) In starved flies, the lactate basal concentration in cortex glia expressing HMGS RNAi was similar to the genotypic control (HMGS: t_12_ = 0.112, P = 0.913). n represents either a group of 40–50 flies analyzed together in a behavioral assay (**a** and **b**), mRNA extracted from a group of 50 flies (**c**) or the response of a single recorded fly (**d-f**). Data are expressed as mean ± s.e.m. with dots as individual values, and analyzed by one-way ANOVA with post hoc testing by Newman-Keuls pairwise comparisons test (**a** and **b**) or by unpaired two-sided t-test (**c-f**). Asterisks refer to the least significant P-value of *post hoc* comparison between the genotype of interest and the genotypic controls (**a** and **b**) or to the P-value of the unpaired t-test comparison (**c-f**). **P<0.01, ns: not significant.

The results that have been presented thus far prompted us to investigate if a sensor system of the cellular energy level could be involved in activating the ketogenic metabolic pathway in cortex glia. We therefore assessed if the major cellular energy sensor AMPK^44^, which has been shown to regulate the activities of the lipase ATGL/Bmm and CPT1 in various models^45–47^, is required specifically in adult cortex glia for K-AM. We observed a strong K-AM impairment when the expression of *AMPKα*, the catalytic subunit of AMPK, was downregulated in adult cortex glia, whereas memory after massed training in fed flies was normal (Fig. 4a). Additionally, K-AM was normal in the absence of RNAi induction, and AMPK*α* was not required in cortex glia for LTM (Extended Data Fig. 6a; see Supplementary Table 4 for sensory controls and also Extended Data Fig. 6b obtained with a second non-overlapping RNAi targeting *AMPKα*). These data show that AMPK is specifically required in cortex glia during starvation to sustain K-AM. We then assessed the involvement of AMPK in FA mobilization from LDs. This first step in KB production is under the control of Bmm (Fig. 2), a known target of AMPK. In the fed condition, expression of AMPK*α* RNAi in cortex glia did not change the mean area of LDs observed in the brain region of cortex glia (Fig. 4b). In contrast, starved flies expressing AMPK*α* RNAi in cortex glia showed larger LDs compared to the genotypic control (Fig. 4b). This demonstrates that during starvation, AMPK activity is required to mobilize FAs stored in LDs in cortex glia.

In *Drosophila*, starvation is known to regulate more than 200 genes at the transcriptional level, with most of them encoding metabolic enzymes^39^. We therefore asked if the genes identified as critical for KB production and transport in cortex glia to sustain K-AM and the genes involved in the neuronal use of KBs are transcriptionally regulated in the fly’s head during starvation. In the heads of wild-type flies, the two enzymes involved in FA mobilization and import to the mitochondria, Bmm and CPT1, respectively, were upregulated during starvation (Fig. 4c), whereas no such increase was observed for other genes involved in KB production and export or for AMPK itself (Extended Data Fig. 6c), this latter result being in agreement with the predominant view that AMPK activation during starvation is achieved by sensing the AMP/ATP ratio^48^. In addition, the expression levels of *ACAT1* and *Sln*, the genes required for K-AM in neurons, did not change in the heads of starved wild-type flies (Extended Data Fig. 6c).

Intriguingly, Bmm and CPT1 are also the two best known downstream effectors of AMPK^45–47^ among all of the genes that we identified in this study. Investigation of the levels of Bmm and CPT1 mRNA in the heads of starved flies expressing AMPK RNAi in adult glia showed that it did not increase after starvation (Fig. 4d). These results demonstrate that AMPK is required in glial cells to mediate the starvation-induced increase in Bmm and CPT1 mRNA levels, and thus to facilitate KB production.

**Fig. 5:**
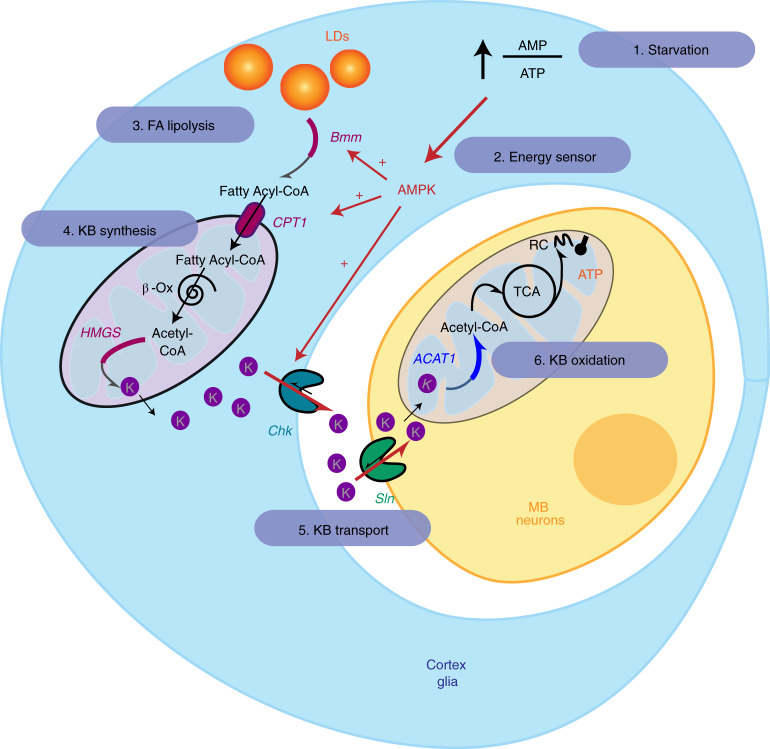
Model of metabolic coupling between glia and neurons during starvation. The AMP/ATP ratio decreases during starvation (1), resulting in the activation of AMPK, the major cellular sensor of energy state. AMPK is required to sustain K-AM formation (2). AMPK is required in cortex glia to increase Bmm and CPT1 expression during starvation and to regulate KB transport by Chk (red arrows). During starvation, FAs are mobilized from the internal stores of cortex glia via the action of the lipase Bmm (3). FAs are then imported into the mitochondria via CPT1 and are subsequently oxidized to generate acetyl-CoA, which is used by HMGS for ketogenesis (4). Eventually, KBs are exported from cortex glia via Chk and taken up by neurons via Sln (5). In neurons, during starvation, KBs are used by ACAT1 to generate acetyl-CoA in the mitochondria for energy (6). TCA, tricarboxylic acid cycle; RC, respiratory chain; K, KB; β-Ox, β-oxidation. The metabolic pathways are symbolized by curved arrows, with the pathway position of the enzyme identified in this study appearing in bold.

Next, we asked if, in addition to its role in regulating KB production, AMPK could also regulate KB export through Chk. To test this hypothesis, we investigated if lactate efflux from cortex glia during KB application, which we found to be dependent on Chk (Fig. 3d), was affected by AMPK downregulation. We observed a decrease in the Laconic ratio (Fig. 4e), as previously observed during bath application of acetoacetate on the brains of starved flies in which cortex glia express the Laconic FRET sensor. When acetoacetate was applied on starved flies expressing AMPK*α* RNAi in the adult cortex glia, the decrease in Laconic ratio was abolished (Fig. 4e), and as AMPK downregulation in cortex glia did not affect lactate basal concentration (Extended Data Fig. 6d), these results showed that the trans-acceleration of lactate efflux by acetoacetate requires AMPK in cortex glia. As this trans-acceleration of lactate efflux after acetoacetate application was specifically impaired by AMPK downregulation and not by downregulation of KB production actors such as HMGS (Extended Data Fig. 6e,f), it suggests that AMPK is directly involved in the regulation of KB export by Chk during starvation independently of KB production in cortex glia. As no change in Chk expression level in the brains of starved flies was detected (Extended Data Fig. 6c), the regulation of Chk-mediated KB transport by AMPK is likely to be at the post-transcriptional level. Altogether, our data reveal that a major function of AMPK in cortex glia during starvation is to adapt the production and export of KBs to MB neurons for K-AM formation, and we have accordingly identified Bmm, CPT1 and Chk as specific targets of AMPK regulation in the pathway either at the transcriptional level for KB production, or at the post-transcriptional level for KB export (Fig. 5).

## Discussion

In this study, we investigated in vivo the metabolic communication between neurons and glia that are used to sustain brain functions during starvation (Fig. 5). We showed that KBs are imported and oxidized by neurons to sustain associative memory formation during starvation. Interestingly, these KBs are provided by a local glia source. By using cell-specific knockdown of enzymes involved in each of the key steps of KB production (that is, FA mobilization, FA mitochondrial import and ketogenesis), we established that cortex glia produce KBs from their own FA internal store and transfer them to neurons. This metabolic communication is critical for K-AM formation in the MB. A combination of behavioural and imaging experiments using the trans-acceleration properties of MCTs allowed us to identify Sln and Chk as the specific MCTs involved in KB transport during starvation in neurons and cortex glia, respectively. Finally, we showed that AMPK, the master energy sensor of the cell, regulates this metabolic communication during starvation by activating KB production and its export by cortex glia.

Our results indicate that the cortex glia mobilize their own internal store of FAs to produce KBs and provide them to neurons. But could this be a more general feature of glial cell types during starvation? We have shown that neither astrocyte-like glia nor ensheathing glia, the two other glial cell types in the *Drosophila* brain that are in close contact with neurons^36^, contribute to KB production to sustain memory formation in neurons during starvation (Extended Data Fig. 4). Thus, the role of LDs as an energy reservoir to sustain neuronal function during starvation seems to be specific to cortex glia. In contrast, its function in other glial cells in which they have been observed^41,42^ should be more related to neuroprotection from damage by reactive oxygen species, as proposed in several *Drosophila* and mammalian studies^41–43^. If the cortex glia in the *Drosophila* brain are the main local provider of KBs, this raises the question of a shared function by glial cells across species, and more specifically in mammals. Even if astrocyte-like glia are the *Drosophila* glial cell type most commonly referred to as the equivalent of mammalian astrocytes^37^, the cortex glia also share some essential morphological features with mammalian astrocytes such as the encompassing of neuronal cell bodies^49^, as well as functions including the modulation of neuronal excitability^50^. Interestingly, mammalian astrocytes present three key points that we have shown to be critical for cortex glia in providing KBs to neurons for sustaining K-AM: (1) they contain LDs^51^; (2) they have (at least in vitro) the metabolic capacity to produce KBs^8,9^; and (3) they express the KB transporter MCT1 (ref. ^7^). Altogether, these arguments suggest that astrocytes in the mammalian brain could provide an additional source of KBs for neurons to sustain neuronal function during starvation. However, at the molecular level, our results show that ketogenesis in *Drosophila* cortex glia depends on a two-step reaction from acetoacetyl-CoA to acetoacetate that relies on HMGS and HMG-lyase, as in the classical path described in the mammalian liver^6,52^. This pathway is different from the one described to occur in vitro in mammalian astrocytes for ketogenesis, which is a one-step reaction catalysed by the reversible enzyme SCOT^8^. Even if succinyl-CoA, the by-product of acetoacetate production by SCOT, is an allosteric inhibitor of HMGS, it is not known if these two pathways used to produce acetoacetate from acetyl-CoA are exclusive, or if they can occur in the same cell in parallel^52^. Further in vivo investigations of the mammalian glia role as a local provider of KBs to neurons, as well as other possible pathways of KB production in *Drosophila* cortex glia, will make it possible to discriminate between experimental set-up bias (in vitro experiments in which only glial cells are present with no neuronal environment), or even differences between mammals and insects.

In insects, the KB concentration increases in the haemolymph during starvation^53^. However, it has still not been clearly established in Drosophila if the fat body (functionally equivalent to the liver) synthesizes and delivers KBs to the haemolymph during starvation. A local provider within the brain such as the cortex glia would be advantageous due to its proximity to neurons, as compared to peripheral organs such as the fat body. This would also circumvent the need to transport KBs across the blood–brain barrier for their uptake by the brain. In addition, a local provider within the brain ensures that the brain will have a KB source with limited competition from other organs, as compared to when KBs are taken up from the haemolymph.

Our results show that AMPK is required in cortex glia to sustain K-AM, suggesting a basal mechanism of KB production and delivery that is activated during starvation (Fig. 5). We identified two well-known downstream effectors of AMPK^45–47^, namely Bmm, the homologue of ATGL^39^, and CPT1 as essential actors of FA mobilization and the subsequent mitochondrial import necessary to sustain K-AM during starvation. We demonstrated that Bmm and CPT1 expression are upregulated during starvation in fly heads and that AMPK in glial cells is required to mediate this transcriptional regulation. The regulation of Bmm and CPT1 by AMPK at the transcriptional level revealed here does not rule out additional post-transcriptional regulations such as phosphorylation of Bmm, as described for ATGL in the activation of its TAG hydrolase activity^45^, and the indirect activation of CPT1 through inhibitory phosphorylation of ACC by AMPK, a mechanism described in various mammalian tissues including the brain^9,47^ that is also conserved across species^54^. Finally, our results demonstrate that, in starved flies, Chk-dependent KB transport is not directly coupled to KB production, whereas it requires AMPK in cortex glia (Fig. 4). Further investigation is required to determine whether the regulation of KB transport via Chk is achieved by regulating Chk activity or Chk trafficking and expression at the membrane, and how AMPK regulates this process.

In mammals, it seems that the brain relies on KB metabolism at two particular times of life: during the postnatal development period^55^; and during ageing, when glucose metabolism becomes impaired^56^. The model proposed in this study of the metabolic coupling between glia and neurons during memory formation based on KB metabolism can provide a framework for further investigations into what occurs during ageing when glucose metabolism is impaired, and how a ketogenic diet might be beneficial in the treatment of neurodegenerative diseases^56^.

## Methods

### Fly strains

*D. melanogaster* flies were raised on standard food medium containing yeast, cornmeal and agar, on a 12 h:12 h light–dark cycle at 18 °C with 60% humidity. The Canton-Special (CS) strain was used as the wild-type strain. All lines were outcrossed for at least three generations with flies carrying a CS wild-type background. For transgene expression in MB neurons, we used the *VT30559-Gal4* line^14^, while the *R54H02-Gal4*, *Alrm-Gal4* and *Mz0709-Gal4* lines were used for specific expression in cortex glia, astrocyte-like glia and ensheathing glia, respectively^36^. Pan-neuronal expression of transgenes was achieved using the *elav-Gal4* line, whereas the *Repo-Gal4* line was used for pan-glial expression. To restrict UAS/GAL4-mediated expression to the adult stage, we used the TARGET system^20^ with the *Tubulin-Gal80*^*ts*^ (*Tub-Gal80*^*ts*^) line as described by Musso et al.^57^. The following inducible driver lines were constructed in the laboratory and have already been described: *Tub-Gal80*^*ts*^*; VT30559-Gal4* (ref. ^14^) and *Tub-Gal80*^*ts*^*; R54H02-Gal4*^15^ and *Tub-Gal80*^*ts*^*; Alrm-Gal4*^15^. Lines that were constructed for this study include *Tub-Gal80*^*ts*^*; Repo-Gal4* and *Tub-Gal80*^*ts*^*; Mz0709-Gal4*. Gal4 activity was released by transferring 0- to 2-day-old adult flies to 30 °C for 2 d.

The following UAS-transgene lines were obtained from the Vienna *Drosophila* Resource Center (VDRC): *UAS-ACAT1 RNAi* *GD7132* (VDRC, v16099), *UAS-Sln RNAi GD1940* (VDRC, v4607)^58^, *UAS-Sln RNAi KK104306* (VDRC, v109464)^58^, *UAS-Bmm RNAi GD5139* (VDRC, v37877)^59^, *UAS-CPT1 RNAi KK100935* (VDRC, v105400)^60^, *UAS-Chk RNAi GD1829* (VDRC, v37139)^25^ and *UAS-HMGS RNAi KK107372* (VDRC, v108245). The following UAS-transgene lines were obtained from the Bloomington *Drosophila* Stock Center (BDSC): *UAS-ACAT1 RNAi HMS03340* (BDSC, 51785), *UAS-Bmm RNAi JF01946* (BDSC, 25926)^59^, *UAS-CPT1 RNAi HMS00040* (BDSC, 34066)^60^, *UAS-HMGS RNAi HMC04928* (BDSC, 57738), *UAS-AMPKα RNAi JF01951* (BDSC, 25931), *UAS-AMPKα* *RNAi* *HMC04979* (BDSC, 57785)^61^ and *UAS-mCD8::RFP* (BDSC, 33219), in addition to the *Chk*^*MB04207*^ line (BDSC, 24296)^26^. In some behavioural experiments (Extended Data Fig. 2c,g), the UAS-Dicer2 transgene (BDSC, 24650) was used in combination with the cortex glia inducible driver (*Tub-Gal80*^*ts*^*; UAS-Dcr2, R54H02-Gal4)* to increase either *Bmm RNAi GD5139* or *CPT1 RNAi KK100935* efficiency, which is an approach that was used successfully in a previous study from our laboratory. Reporter lines used in this study include *CRIMIC Sln-T2A-Gal4* from BDSC (79274)^29^ and *Chk-Gal4*^*MI15450*^ (ref. ^26^), provided by J. Sierralta. The *UAS-Laconic* line was generated previously in our research group^22^.

**Extended Data Fig. 7:**
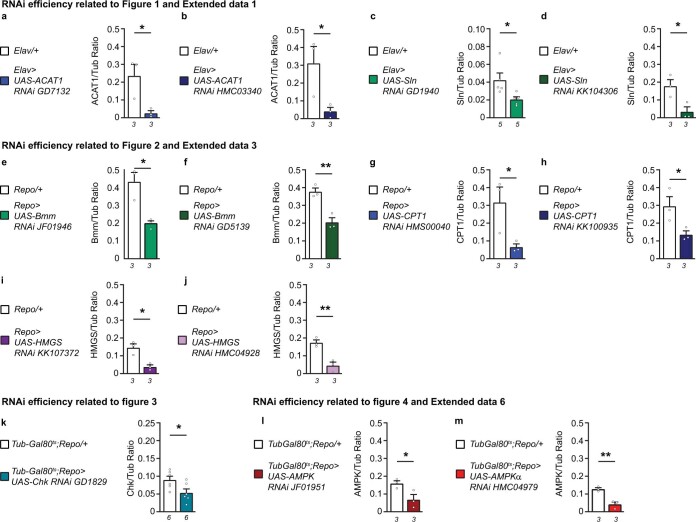
Validation of RNAi efficiency of the RNAi lines used in this study. (**a**–**d**) Using the pan-neuronal driver *elav* to drive constitutive expression of the specified RNAi, we observed a significant reduction of the targeted mRNA level in fly heads: ACAT1 RNAi GD7132 (**a**) t_4_ = 3,182, p = 0.033; ACAT1 RNAi HMC03340 (**b**) t_4_ = 2,790, p = 0.049); Sln RNAi GD1940 (**c**) t_8_ = 2,600, p = 0.032 and Sln RNAi KK104306 (**d**) t_4_ = 3,227, p = 0.032. (**e-j**) Using the pan-glial driver *repo* to drive constitutive expression of the specified RNAi, we observed a significant reduction of the targeted mRNA level in fly heads: Bmm RNAi JF0146 (**e**) t_4_ = 4,329, p = 0.012; Bmm RNAi GD5139 (**f**) t_4_ = 5,309, p = 0.006; CPT1 RNAi HMS00040 (**g**) t_4_ = 2,832, p = 0.047; CPT1 RNAi KK100935 (**h**) t_4_ = 2,784, p = 0.049; HMGS RNAi KK107372 (**i**) t_4_ = 4,533, p = 0.011 or HMGS RNAi HMC04928 (**j**) t_4_ = 5,295, p = 0.006. (**k-m**) Because constitutive expression of Chk RNAi or AMPK RNAi in glial cells was lethal, the RNAi efficiency was assessed by measuring the targeted mRNA level in fly heads expressing the RNAi in adult glial cells using the pan-glial inducible driver *Tub-Gal80*^*ts*^*; Repo-Gal4*: Chk RNAi GD1829 (**k**) t_10_ = 2.43, P = 0.035; AMPK RNAi JF01951 (**l**) t_4_ = 2.84, P = 0.047 and AMPK RNAi HMC04979 (**m**) t_4_ = 5.72, P = 0.0046. n represents mRNA extracted from heads of a group of 50 flies. Results are shown as ratios to the reference gene tubulin and data are expressed as mean ± s.e.m. with dots as individual values, and analyzed by unpaired two-sided t-test. Asterisks refer to the P-value of the unpaired t-test comparison. **P<0.01, *P<0.05.

For each UAS-RNAi line listed above, the efficiency of each RNAi construction to decrease mRNA level of the targeted gene was confirmed following the protocol detailed in ‘Quantitative PCR analyses’. The results are presented in Extended Data Fig. 7.

### Olfactory conditioning and memory test

The behavioural experiments, including sample sizes, were conducted similarly to previous studies from our research group^14–16^. For all experiments, training and testing were performed at 25 °C and 80% humidity; after conditioning, flies were kept at 18 °C until testing. Briefly, groups of approximately 30–40 flies were subjected to one of the following olfactory conditioning protocols: five consecutives associative training cycles (5× massed), or five associative cycles spaced by 15-min inter-trial intervals (5× spaced). Custom-built barrels allowing parallel training of up to six groups were used for conditioning. Throughout the conditioning protocol, each barrel was plugged into a constant airflow at 2 l min^−1^. The sequence of one conditioning cycle consisted of an initial 90-s period of non-odorized airflow, followed by 60 s of the conditioned odour paired with 12 pulses of 1.5 s, 60-V electric shocks. Then, after 45 s of non-odorized airflow, the second odour was presented for 60 s without electroshocks, followed by 45 s of non-odorized airflow. The odorants, 3-octanol (>95% purity; Fluka 74878, Sigma-Aldrich) and 4-methylcyclohexanol (99% purity; Fluka 66360), were diluted in paraffin oil at 0.360 mM and 0.325 mM, respectively, and were alternately used as conditioned stimuli.

The memory test was performed in a T-maze apparatus, typically after 24 h of massed or spaced training. Flies were exposed simultaneously to both odorants for 1 min in the dark. The PI was calculated as the number of flies attracted to the unconditioned odour minus the number of flies attracted to the conditioned odour, divided by the total number of flies in the experiment, and the resulting number was multiplied by 100. A single memory PI value is the average of the scores from two groups of flies of the same genotype trained with either 3-octanol or 4-methylcyclohexanol as the conditioning stimulus. The indicated ‘*n*’ is the number of independent PI values for each genotype.

In the fed condition, experiments were conducted similarly to other studies from our research group^14,15^. Briefly, to achieve RNAi induction, 1- to 2-day-old flies were kept at 30 °C for 2 d until conditioning. The non-induced control flies, in which RNAi expression is inhibited, were kept at 18 °C.

The starvation procedure is similar to our previously used procedure before appetitive memory conditioning^62,63^. To achieve RNAi induction in the starved condition, 1- to 2-day-old flies were kept at 30 °C for 2 d until conditioning; then, 16 h before conditioning, the flies were transferred to starvation bottles containing only a filter paper soaked with 6.8 ml of mineral water (Evian). Non-induced starved control flies were deprived of food for 21 h at 25 °C. During the 24-h storage period after conditioning, flies were kept in starvation bottles at 18 °C.

Olfactory avoidance and shock avoidance tests were conducted similarly to previous studies from our research group as in work by ref. ^15^ except that flies were kept at 30 °C for 2 d and food deprived for 16 h before testing.

### In vivo lactate imaging

As in all previous imaging works from our laboratory^14,15,62^, in vivo imaging experiments were carried out in female flies due to their larger size, which makes surgery easier. Briefly, female flies carrying a *Tub-Gal80*^*ts*^*, UAS-Laconic;VT30559-GAL4* or *Tub-Gal80*^*ts*^*, UAS-Laconic;R54H02-GAL4* construct were crossed to CS males or to males carrying the appropriate UAS-RNAi (*UAS-Sln RNAi GD1940* for MB neurons imaging, and *UAS-Chk RNAi GD1829*, *UAS-AMPKα RNAi JF01951* or *UAS-HMGS RNAi KK107372* for cortex glia imaging). Crosses for imaging experiments were raised at 23 °C to avoid expression of the RNAi during development. The 1- to 2-day-old adult progeny were induced for 3 d at 30 °C. As with the memory assays, induced starved flies were deprived of food for 16 h before imaging experiments. A single fly was affixed to a plastic coverslip using a non-toxic dental glue (Protemp II 3 M ESPE). Then, 90 μl of an artificial haemolymph solution was added on top of the coverslip. The composition of the artificial haemolymph solution for fed flies, which we will refer to as ‘fed flies saline’ solution, was: 130 mM NaCl (Sigma, S9625), 5 mM KCl (Sigma, P3911), 2 mM MgCl_2_ (Sigma, M9272), 2 mM CaCl_2_ (Sigma, C3881), 5 mM d-trehalose (Sigma, T9531), 30 mM sucrose (Sigma, S9378) and 5 mM HEPES-hemisodium salt (Sigma, H7637). The composition of the artificial haemolymph solution for starved flies, which we will refer to as ‘starved flies saline’ solution, was the same as the solution for fed flies except that it contained 36 mM ribose (Sigma, W379301) instead of sucrose and trehalose. Surgery was performed as previously described^14,15,62^ to expose the brain for optical imaging. At the end of the surgery, a fresh drop of 90 μl of the appropriate saline solution was applied on the aperture in the fly head’s cuticle. Two-photon imaging was performed on a Leica TCS-SP5 upright microscope equipped with a ×25, 0.95-NA water-immersion objective. Two-photon excitation of mTFP was achieved using a Mai Tai DeepSee laser tuned to 825 nm. Then, 512 × 256 images were acquired at a frame rate of one image every 2 s, and the entire duration of each recording was 400 s. For the trans-acceleration experiments or the lactate bath application experiment, acetoacetate (Sigma, A8509) or l-lactate (Sigma, L7022), respectively, was diluted into the appropriate saline solution depending on the feeding status of the fly at a stock concentration of 100 mM. After 180 s of baseline acquisition, 10 μl of acetoacetate stock solution or l-lactate stock solution was added to the 90-μl saline solution drop on top of the brain, for a final concentration of 10 mM.

For the lactate saturation experiments, sodium azide (Sigma, 71289) was diluted into the appropriate saline solution depending on the feeding status of the fly at a stock concentration of 50 mM. After  180s of baseline acquisition, 10 μl of the sodium azide stock solution was added to the 90 μl saline solution drop on top of the brain, for a final concentration of 5 mM.

Image analysis was performed using a custom-written MATLAB script^64^. Regions of interest (ROIs) were delimited by hand around each visible region of Kenyon cell somas or in the cortex glia in the proximity of the MB calyx. The average intensity levels of mTFP and Venus channels over each ROI were calculated over time after background subtraction. The FRET Laconic ratio was calculated by dividing mTFP intensity by Venus intensity. In addition, for lactate saturation experiments, lactate traces with sodium azide treatment were normalized to the final plateau value (defined as the 100-s-long time window starting 180 s after sodium azide application), which corresponds to a fully bound (that is, saturated) state of the sensor. The baseline lactate concentration was then estimated as the average Laconic ratio level during the baseline recording before acquisition. Traces from all hemispheres were pooled.

### Lipid droplet staining and image analysis

Neutral LDs were detected with a non-polar fluorescence probe, BODIPY 493/503 (Sigma, D3922). BODIPY staining was carried out according to a previously published protocol^58^. Female flies carrying the *Tub-Gal80*^*ts*^*;R54H02* cortex glia-specific driver were crossed with males carrying the specified UAS-RNAi or with CS males. For behavioural experiments, crosses were raised at 18 °C and the 1- to 2-day-old adult progeny were induced for 2 d at 30 °C and transferred to starvation bottles 16 h before dissection. Groups of flies from the same induced bottle were divided into fed and starved conditions. Before dissection, flies were anaesthetized on ice. Brains were dissected on ice in 1× PBS (Sigma, P4417) and then fixed for 30 min in 4% paraformaldehyde (Electron Microscopy Sciences, 15710) in 1× PBS at room temperature. After three washes in 1× PBS, brains were incubated for 30 min with 1 μM BODIPY 493/503 in the dark. After three washes in 1× PBS, brains were mounted using Prolong Mounting Medium (Life Technologies, P36965). Mounting and image acquisition were carried out on the same day. Next, 1,024 × 1,024 images were acquired with a Nikon A1R confocal microscope equipped with a ×100/1.40 oil-immersion objective in the cortex region, in the proximity of the MB calyx. Confocal excitation of BODIPY was achieved using a laser tuned to 488 nm. Confocal *z*-stacks of LDs were imported into Fiji (ImageJ 1.52p)^65^ and CellProfiler 3.1.9 Analyst software^66^ for further analyses. Briefly, a single plane in the cortex region was selected and converted into an 8-bit greyscale image. A specific rectangular area (84 × 80 μm) in close proximity to the calyx was selected for analysis. Because BODIPY labels the plasma membrane in addition to LDs, resulting in a bright spot-like staining over the plasma membrane’s more uniform and dimmer staining, it was necessary to perform thresholding of the image to remove the plasma membrane signal. For a non-arbitrary determination of the threshold needed to keep only the LD BODIPY staining, we set up the following procedure. For each ROI, the pixel intensity histogram of the greyscale ROI was exported from Fiji and a Gaussian fit was performed in Prism 8.0 (GraphPad); *x* < 3 and *x* > 40 values were excluded from the fit to avoid extreme values such as black pixels. The mean and s.d. parameters of this Gaussian fit were extracted and used to calculate the threshold value, which was set for all images to: ((mean intensity + 4 × s.d.)/255). This threshold was applied for further analyses using CellProfiler 3.1.9 Analyst. For each ROI, after thresholding and applying a size-limit object filter (0.37–1.5 μm^2^) based on previous LD data in the literature^67,68^, object detection and counting were performed to identify LDs. For each ROI, the area of each identified LD was calculated and expressed in μm^2^ and used to calculate the mean area of LDs per ROI. For each brain, an ROI from each hemisphere was analysed and the results from both hemispheres were averaged. For a few cases in which only one hemisphere could be properly visualized for quantification, only one ROI was used for analysis. The indicated ‘*n*’ corresponds to the number of brains analysed.

### Quantitative PCR analyses

Quantitative PCR analyses to assess the effect of starvation on specific gene mRNA levels were conducted similarly to previous studies from our research group^69,62^. To assess the efficiency of each RNAi used in this study, female flies carrying the *elav-Gal4* pan-neuronal driver or the *repo-Gal4* pan-glial driver were crossed with males carrying the specified UAS-RNAi or with CS males. Fly progeny were reared at 25 °C throughout their development. Then, 0- to 1-day-old flies were transferred to fresh food for 1 d before RNA extraction. As the *UAS-Chk RNAi GD1829*, *UAS-AMPK RNAi JF01951* and *UAS-AMPK RNAi HMC04979* lines exhibit lethality at larval stages when expressed constitutively in glial cells^25,70^, these specific *UAS-RNAi* lines were crossed with the inducible *Tub*-*Gal80*^*ts*^*;Repo-Gal4 line*, reared at 18 °C throughout their development, and the adult progeny were induced for 4 d at 30 °C before extraction. To assess the effect of starvation on the level of mRNA of specific genes, CS flies were reared at 25 °C throughout their development. Then, 0- to 1-day-old flies were transferred to fresh food for 1 d, and were then separated into a fed and a starved group (21 h of food deprivation at 25 °C) before RNA extraction. To assess the effect of AMPK*α* on the mRNA levels of *Bmm* and *CPT1*, female flies carrying the *Tub-Gal80*^*ts*^*;Repo-Gal4* pan-glial driver were crossed with males carrying the *UAS-RNAi AMPKα JF01951* or with CS males. Crosses were raised at 18 °C and the 1- to 2-day-old adult progeny were induced for 4 d at 30 °C and transferred to starvation bottles 16 h before extraction. Groups of flies from the same induced bottle were divided into fed and starved conditions.

Except for ACAT1 and HMGS genes (see detail below), RNA extraction and cDNA synthesis were done as in refs. ^69,62^ using the same reagent: the RNeasy Plant Mini Kit (Qiagen), RNA MinElute Cleanup kit (Qiagen), oligo(dT)20 primers and the SuperScript III First-Strand kit (Life Technologies). Because *ACAT1* and *HMGS* comprise a single coding exon and no intronic sequences, preparations underwent an additional step after mRNA extraction and before cDNA synthesis of DNase I treatment (BioLabs) for 15 min at 37 °C and subsequent DNase heat inactivation with EDTA (10 mM), to avoid any contamination from genomic DNA. The level of cDNA for each gene of interest was compared against the level of the α-Tub84B (Tub, CG1913) reference cDNA. Amplification was performed using a LightCycler 480 (Roche) and the SYBR Green I Master mix (Roche). Reactions were carried out in triplicate. The specificity and size of amplification products were assessed by melting curve analyses. Expression relative to the reference was expressed as a ratio (2^−^^ΔCp^, where Cp is the crossing point). The complete sequence of each pair of primers used for each gene is reported in Supplementary Table 5.

### Immunohistochemistry experiments

Female flies carrying *CRIMIC Sln-T2A-Gal4* (ref. ^29^) or *Chk-Gal4*^*MI15450*^ (ref. ^26^) were crossed to male flies carrying the *UAS-mCD8::RFP* construct. Before dissection, 2- to 4-day-old female flies were fixed in 4% paraformaldehyde in PBST (PBS containing 1% of Triton X-100) at 4 °C overnight. Fly brains were dissected on ice in PBS solution, fixed for 1h in 4% paraformaldhehyde PBST and rinsed three times for 20 min in PBST. Then, brains were blocked with 2% BSA in PBST for 2 h. Next, samples were incubated with primary antibodies in the blocking solution (2% BSA in PBST) at 4 °C overnight. The following primary antibodies were used: a 1:250 dilution of rabbit anti-RFP (Clontech, 632496), a 1:100 dilution of mouse anti-nc82 (DSHB, nc82) and a 1:100 dilution of mouse anti-Wrapper (DSHB, Wrapper). The following day, brains were rinsed three times for 20 min with PBST and then incubated for 3 h at room temperature with secondary antibodies diluted in blocking solution. The following secondary antibodies were used: a 1:400 dilution of anti-mouse conjugated to Alexa Fluor 488 (Invitrogen, A11029), a 1:400 dilution of anti-rabbit conjugated to Alexa Fluor 594 (Invitrogen, A11037) and a 1:400 dilution of anti-mouse conjugated to Alexa Fluor 633 (Invitrogen, A-21126). Brains were then rinsed once in PBST for 20 min, and twice in PBS for 20 min. After rinsing, brains were mounted using Prolong Mounting Medium (Invitrogen). Acquisitions were made with a Nikon A1R confocal microscope, with either a ×40/1.15 water-immersed objective or a 100x/1.40 oil-immersion objective.

### Statistical analysis

Statistical parameters including the definitions and exact value of *n*, deviations and *P* values are reported in the figures and corresponding legends. Data are expressed as the mean ± s.e.m. with dots as individual values corresponding to a group of 40–50 flies analysed together in a behavioural assay, to the response of a single recorded fly for lactate imaging and to one BODIPY-stained brain for LD experiments, and to one mRNA extraction from heads of a group of 50 flies for RT–qPCR experiments. Statistical analysis was performed using GraphPad Prism 8.0. Comparisons between two groups were performed by unpaired two-sided Student’s *t*-test, with results given as the value *t*_*x*_ of the *t-*distribution, where *x* is the number of degrees of freedom. Comparisons among three groups were performed by one-way ANOVA with post hoc testing by the Newman–Keuls pairwise comparisons test between the experimental group and its controls (significance is indicated when *P* < 0.05). ANOVA results are given as the value of the Fisher distribution F_(*x*,*y*)_, where *x* is the number of degrees of freedom numerator and *y* is the total number of degrees of freedom denominator. Asterisks in each figure refer to the least-significant post hoc comparison between the genotype of interest and the genotypic controls. The nomenclature used corresponds to *P < 0.05, **P < 0.01, ***P < 0.001, ****P < 0.0001; NS, *P* > 0.05. Figures were created using Adobe Illustrator CS6.

### Reporting Summary

Further information on research design is available in the Nature Research Reporting Summary linked to this article.

## Supplementary information

Supplementary Information Supplementary Tables 1–5

Reporting Summary

## Data Availability

No datasets that require mandatory deposition into a public database were generated during the current study. Any data generated and/or analysed during the current study are available from the corresponding author on reasonable request.

## Code availability

Imaging analysis of in vivo lactate imaging was performed using a custom-written MATLAB script modified from Plaçais et al.^14^ available on Zenodo^64^ at 10.5281/zenodo.5791642.
