# Supplementary material for: Glia fuel neurons with locally synthesized ketone bodies to sustain memory under starvation
Source: Nat Metab. 2022 Feb 17;4(2):213–24. doi: 10.1038/s42255-022-00528-6 (PMC8885408; doi:10.1038/s42255-022-00528-6)
Supplement: Supplementary file 1 — Supplementary Tables 1–5 [file 42255_2022_528_MOESM1_ESM.pdf]

nature portfolio

https://doi.org/10.1038/s42255-022-00528-6

Supplementary information

Glia fuel neurons with locally synthesized ketone bodies to sustain memory under starvation

In the format provided by the authors and unedited

Supplementary Table 1. Sensory control experiments related to Fig. 1 and Extended data 1

	Shock Avoidance			Naive odor avoidance					
				Octanol			Methylcyclohexanol		
Genotypes	Mean ± s.e.m.	n	Statistics	Mean ± s.e.m.	n	Statistics	Mean ± s.e.m.	n	Statistics
TubGal80^{ts};VT30559/+	0.42±0.09	8		0.73±0.04	8		0.67±0.06	8	
TubGal80^{ts};VT30559> UAS-ACAT1 RNAi GD7132	0.46±0.06	8	$F_{2,21}$=0.48 p=0.63	0.70±0.03	8	$F_{2,21}$=0.17 p=0.85	0.53±0.05	8	$F_{2,21}$=1.09 p=0.36
UAS-ACAT1 RNAi GD7132/+	0.53±0.08	8		0.70±0.05	8		0.58±0.09	8	
TubGal80^{ts};VT30559/+	0.58±0.06	8		0.49±0.10	8		0.72±0.06	8	
TubGal80^{ts}; VT30559> UAS-ACAT1 RNAi HMC03340	0.47±0.07	8	$F_{2,21}$=0.41 p=0.67	0.36±0.11	8	$F_{2,21}$=1.87 p=0.18	0.71±0.03	8	$F_{2,21}$=0.27 p=0.77
UAS-ACAT1 RNAi HMC03340/+	0.49±0.13	8		0.23±0.08	8		0.67±0.05	8	
TubGal80^{ts};VT30559/+	0.46±0.07	8		0.85±0.03	8		0.81±0.04	8	
TubGal80^{ts};VT30559> UAS-Silnoon RNAi GD1940	0.47±0.10	8	$F_{2,21}$=0.16 p=0.85	0.79±0.05	8	$F_{2,21}$=0.36 p=0.70	0.80±0.03	8	$F_{2,21}$=0.03 p=0.97
UAS-Silnoon RNAi GD1940/+	0.52±0.06	8		0.81±0.05	8		0.81±0.05	8	
TubGal80^{ts};VT30559/+	0.27±0.08	8		0.65±0.05	8		0.76±0.03	8	
TubGal80^{ts}; VT30559> UAS-Silnoon RNAi KK104306	0.40±0.08	8	$F_{2,21}$=1.27 p=0.30	0.63±0.06	8	$F_{2,21}$=2.93 p=0.075	0.80±0.03	8	$F_{2,21}$=0.20 p=0.82
UAS-Silnoon RNAi KK104306/+	0.45±0.10	8		0.45±0.08	8		0.75±0.08	8	

Expression of the different RNAi constructs used in Figure 1 and extended data 1 in MB neurons did not have any significant effect on olfactory acuity or the avoidance of electric shocks. n represents a group of 40-50 flies analyzed together in a behavioral assay. Data are expressed as mean ± s.e.m. and analyzed by one-way ANOVA. *P*-value of the ANOVA is indicated.

Supplementary Table 2. Sensory control experiments related to Fig. 2 and Extended data 3

	Shock Avoidance			Naive odor avoidance					
				Octanol			Methylcyclohexanol		
Genotypes	Mean ± s.e.m.	n	Statistics	Mean ± s.e.m.	n	Statistics	Mean ± s.e.m.	n	Statistics
TubGal80ts;R54H02/+	0.52±0.08	8		0.68±0.09	8		0.73±0.05	8	
TubGal80ts;R54H02> UAS-Bmm RNAi JF01946	0.38±0.07	8	$F_{2,21}$=0.73 p=0.50	0.80±0.05	8	$F_{2,21}$=1.25 p=0.31	0.64±0.05	8	$F_{2,21}$=0.78 p=0.47
UAS-Bmm RNAi JF01946/+	0.50±0.11	8		0.80±0.05	8		0.72±0.06	8	
TubGal80ts;Dcr2, R54H02/+	0.60±0.05	12		0.47±0.11	8		0.82±0.04	8	
TubGal80ts;Dcr2, R54H02 > UAS-Bmm RNAi GD5139	0.65±0.08	12	$F_{2,33}$=0.99 p=0.38	0.37±0.12	8	$F_{2,21}$=1.354 p=0.28	0.80±0.04	8	$F_{2,21}$=1.86 p=0.18
UAS-Bmm RNAi GD5139/+	0.73±0.07	12		0.24±0.06	8		0.70±0.06	8	
TubGal80ts;R54H02/+	0.43±0.09	12		0.38±0.06	8		0.43±0.07	8	
TubGal80ts;R54H02> UAS-CPT1 RNAi HMS00040	0.49±0.11	12	$F_{2,33}$=0.08 p=0.92	0.38±0.09	8	$F_{2,21}$=0.13 p=0.88	0.45±0.05	8	$F_{2,21}$=0.13 p=0.88
UAS-CPT1 RNAi HMS00040/+	0.47±0.10	12		0.43±0.06	8		0.47±0.06	8	
TubGal80ts;Dcr2, R54H02/+	0.41±0.07	8		0.42±0.07	8		0.39±0.11	8	
TubGal80ts;Dcr2, R54H02> UAS-CPT1 RNAi KK100935	0.46±0.10	8	$F_{2,21}$=0.33 p=0.72	0.35±0.03	8	$F_{2,21}$=0.91 p=0.42	0.38±0.13	8	$F_{2,21}$=0.027 p=0.97
UAS-CPT1 RNAi KK100935/+	0.35±0.10	8		0.31±0.06	8		0.41±0.10	8	
TubGal80ts;R54H02/+	0.48±0.04	10		0.81±0.04	8		0.78±0.06	8	
TubGal80ts;R54H02> UAS-HMGS RNAi KK107372	0.45±0.08	10	$F_{2,27}$=0.17 p=0.85	0.57±0.08	8	$F_{2,21}$=4.25 p=0.03 *	0.63±0.09	8	$F_{2,21}$=1.55 p=0.24
UAS-HMGS RNAi KK107372/+	0.44±0.04	10		0.61±0.06	8		0.78±0.05	8	
TubGal80ts; R54H02/+	0.40±0.10	9		0.66±0.05	8		0.62±0.09	8	
TubGal80ts;R54H02> UAS-HMGS RNAi HMC04928	0.55±0.05	9	$F_{2,24}$=0.85 p=0.44	0.65±0.05	8	$F_{2,21}$=0.56 p=0.058	0.50±0.12	8	$F_{2,21}$=0.24 p=0.79
UAS-HMGS RNAi HMC04928/+	0.46±0.09	9		0.73±0.06	8		0.53±0.16	8	

Expression of the different RNAi constructs used in Figure 2 and extended data figure 3 in cortex glia did not have any significant effect on olfactory acuity or the avoidance of electric shocks. n represents a group of 40-50 flies analyzed together in a behavioral assay. Data are expressed as mean ± s.e.m. and analyzed by one-way ANOVA. *P*-value of the ANOVA is reported. Post hoc testing by Newman-Keuls pairwise comparisons test P is done only if the P-value of the ANOVA is significant (p<0.05). Then the P-value indicated is the lowest one obtained from the two pairwise comparisons between the RNAi expressing flies and their driver (*tubulin-GAL80ts; VT30559/+* or *tubulin-GAL80ts; R54H02/+*) or effector (*UAS-…RNAi/+*) controls. *: result of the post-hoc comparison: *TubGal80ts;R54H02>UAS-HMGS RNAi KK107372* vs *UAS-HMGS RNAi KK107372/+* p-value=non-significant.

Supplementary Table 3. Sensory control experiments related to Fig. 3 and Extended data 5

	Shock Avoidance			Naive odor avoidance					
				Octanol			Methylcyclohexanol		
Genotypes	Mean ± s.e.m.	n	Statistics	Mean ± s.e.m.	n	Statistics	Mean ± s.e.m.	n	Statistics
TubGal80^{ts};R54H02/+	0.33±0.07	10	$F_{2,27}$=0.06 p=0.95	0.41±0.07	8	$F_{2,21}$=0.18 p=0.83	0.40±0.09	8	$F_{2,21}$=0.42 p=0.66
TubGal80^{ts};R54H02> UAS-Chk RNAi GD1829	0.36±0.06	10		0.46±0.08	8		0.50±0.08	8	
UAS-Chk RNAi GD1829/+	0.33±0.07	10		0.40±0.06	8		0.43±0.08	8	
+/w^{1118}	0.26±0.06	12	t_{21}=0.55 p=0.59	0.25±0.08	8	t_{14}=0.31 p=0.76	0.45±0.11	8	t_{14}=0.02 p=0.99
+/ w^{1118};+/Chk^{MB04207}	0.32±0.09	11		0.28±0.04	8		0.45±0.11	8	

Expression of the Chk RNAi in cortex glia did not have any significant effect on olfactory acuity or the avoidance of electric shocks. Heterozygous *w^{1118}/+; Chk^{MB04207}/+* female flies had normal olfactory acuity and avoidance to electric shocks compared to control *w^{1118}/+;+* female flies. n represents a group of 40-50 flies analyzed together in a behavioral assay. Data are expressed as mean ± s.e.m. and analyzed by unpaired two-sided t-test for comparison between *w^{1118}/+; Chk^{MB04207}/+* and *w^{1118}/+;+* female flies the *P*-value of the t-test is indicated.

Supplementary Table 4. Sensory control experiments related to Fig. 4 and Extended data 6

	Shock Avoidance			Naive odor avoidance					
				Octanol			Methylcyclohexanol		
Genotypes	Mean ± s.e.m.	n	Statistics	Mean ± s.e.m.	n	Statistics	Mean ± s.e.m.	n	Statistics
TubGal80^{ts};R54H02/+	0.62±0.07	8	$F_{2,21}$=0.31 p=0.74	0.53±0.11	8	$F_{2,21}$=0.64 p=0.54	0.76±0.06	8	$F_{2,21}$=0.24 p=0.79
TubGal80^{ts};R54H02> UAS-AMPK RNAi JF01951	0.60±0.11	8		0.43±0.09	8		0.70±0.06	8	
UAS-AMPK RNAi JF01951/+	0.53±0.08	8		0.56±0.04	8		0.72±0.06	8	
TubGal80^{ts};R54H02/+	0.34±0.09	10	$F_{2,27}$=0.31 p=0.73	0.50±0.09	8	$F_{2,21}$=0.04 p=0.96	0.53±0.07	8	$F_{2,21}$=0.40 p=0.68
TubGal80^{ts};R54H02> UAS-AMPK RNAi HMC04979	0.43±0.07	10		0.46±0.14	8		0.50±0.11	8	
UAS-AMPK RNAi HMC04979/+	0.38±0.08	10		0.51±0.12	8		0.60±0.07	8	

Expression of the different RNAi constructs used in Figure 4 and Extended data 9 in cortex glia did not have any significant effect on olfactory acuity or the avoidance of electric shocks. n represents a group of 40-50 flies analyzed together in a behavioral assay. Data are expressed as mean ± s.e.m. and analyzed by one-way ANOVA and the *P*-value of the ANOVA is indicated.

Supplementary Table 5. Primers sequences.

Gene	Related to Figure:	Forward primer	Reverse primer
ACAT1	Ext data 6 and 7	ATTGCGAAGACCGATGTCCAG	GCAGCATACATTGGTGGGC
Sln	Ext data 6 and 7	CTGCTGCACCTCCTCCTAAC	ATGCACGGTGCTTGTCAAAG
Bmm	Fig. 4 and Ext. data 7	CACTTCGATGCCACAGGAC	GACATCGTCCTCTAGCGGTG
CPT1	Fig. 4	CACCAGGAACTGCAGCCTAT	CATACTGCCAGGAGCACAGA
	Ext. data 7	ACCATTGAGTCATCCGCCTG	CATCCTGGTAGCCCAACTGG
HMGS	Ext data 6 and 7	AAGAAATCGCTGCTGCTG	CCGCTAATCAACAGAGACAC
Chk	Ext data 6	CATCTACCGCAACTCGATGA	GTGTCCACAAAGTTGTCATACCA
	Ext data 7	CTCTACATTGTTTTTGTTCATTTGGT	CCGACATCCGAAATCAACTC
AMPKα	Ext data 6 and 7	CGCACATCATCAAGTTGTACC	TTGGTGCTCCTGCAGCTT

Table reporting all the primers used to investigate the effect of starvation on the expression of the targeted gene (Fig.4 and Extended data 6) and/or to assess the RNAi efficiency (Extended data 7).
