# Supplementary material for: Glia fuel neurons with locally synthesized ketone bodies to sustain memory under starvation
Source: Nat Metab. 2022 Feb 17;4(2):213–24. doi: 10.1038/s42255-022-00528-6 (PMC8885408; doi:10.1038/s42255-022-00528-6)
Supplement: Supplementary file 2 — Reporting Summary [file 42255_2022_528_MOESM2_ESM.pdf]

nature portfolio

Corresponding author(s): Alice Pavlowsky and Thomas Preat

Last updated by author(s): Dec 20, 2021

Reporting Summary

Nature Portfolio wishes to improve the reproducibility of the work that we publish. This form provides structure for consistency and transparency in reporting. For further information on Nature Portfolio policies, see our Editorial Policies and the Editorial Policy Checklist.

Statistics

For all statistical analyses, confirm that the following items are present in the figure legend, table legend, main text, or Methods section.

n/a	Confirmed	
☐	☒	The exact sample size (*n*) for each experimental group/condition, given as a discrete number and unit of measurement
☐	☒	A statement on whether measurements were taken from distinct samples or whether the same sample was measured repeatedly
☐	☒	The statistical test(s) used AND whether they are one- or two-sided<br>*Only common tests should be described solely by name; describe more complex techniques in the Methods section.*
☒	☐	A description of all covariates tested
☐	☒	A description of any assumptions or corrections, such as tests of normality and adjustment for multiple comparisons
☐	☒	A full description of the statistical parameters including central tendency (e.g. means) or other basic estimates (e.g. regression coefficient) AND variation (e.g. standard deviation) or associated estimates of uncertainty (e.g. confidence intervals)
☐	☒	For null hypothesis testing, the test statistic (e.g. *F*, *t*, *r*) with confidence intervals, effect sizes, degrees of freedom and *P* value noted<br>*Give P values as exact values whenever suitable.*
☒	☐	For Bayesian analysis, information on the choice of priors and Markov chain Monte Carlo settings
☒	☐	For hierarchical and complex designs, identification of the appropriate level for tests and full reporting of outcomes
☒	☐	Estimates of effect sizes (e.g. Cohen's *d*, Pearson's *r*), indicating how they were calculated

Our web collection on statistics for biologists contains articles on many of the points above.

Software and code

Policy information about availability of computer code

Data collection	n vivo Lactate imaging experiments were collected with the LAS AF Version 2.7.3 (Leica Microsystems).<br>Images of immuno-labelled brains and of brain's Lipid droplets were collected with NIS-Element AR 4.40.00 (Nikon).<br>qRT-PCR data collection and processing was performed using LightCycler 480SW 1.5 (Roche Life Science).
Data analysis	Statistical analysis was done using GraphPad Prism 8 (GraphPad Software). LD analysis was done using Fiji (ImageJ 1.52p) and CellProfiler 3.1.9 Analyst Software. Lactate imaging analysis was done using a custom-written MatLab R2019b script available on Zenodo (doi: 10.5281/zenodo.5791642). Figures were made using Adobe illustrator CS6.

For manuscripts utilizing custom algorithms or software that are central to the research but not yet described in published literature, software must be made available to editors and reviewers. We strongly encourage code deposition in a community repository (e.g. GitHub). See the Nature Portfolio guidelines for submitting code & software for further information.

Data

Policy information about availability of data

All manuscripts must include a data availability statement. This statement should provide the following information, where applicable:

- Accession codes, unique identifiers, or web links for publicly available datasets
- A description of any restrictions on data availability
- For clinical datasets or third party data, please ensure that the statement adheres to our policy

No data sets that require mandatory deposition into a public database were generated during the current study. Any data generated and/or analyzed during the current study are available from the corresponding author on reasonable request.

Field-specific reporting

Please select the one below that is the best fit for your research. If you are not sure, read the appropriate sections before making your selection.

☒ Life sciences ☐ Behavioural & social sciences ☐ Ecological, evolutionary & environmental sciences

For a reference copy of the document with all sections, see nature.com/documents/nr-reporting-summary-flat.pdf

Life sciences study design

All studies must disclose on these points even when the disclosure is negative.

Sample size	Sample size choice was based on previous studies from our group such as Plaçais et al. Nat. Com. 2017 for behavior and live imaging experiment and Silva et al. J. Neurogenet. 2020 for RT-qPCR experiments, or based on standard publication such as Van Den Brink et al. PLoS Genet. 2017 for lipid droplets staining but not predetermined by a statistical method.
Data exclusions	No data was excluded
Replication	All quantification were performed unblinded. Statistical parameters including the definitions and exact value of n (e.g. number of group of flies (behavior experiments), number of brains (in vivo live-imaging and LD staining), number of experiments replication (RT-qPCR), deviations and p values are reported in the figures and corresponding figure legends. Statistical analysis was carried out using Prims8 (Graph Pad Software). Comparisons between two groups were performed by unpaired two-sided Student's t-test, with results given as the value tx of the t distribution, where x is the number of degrees of freedom. Comparisons among three groups were performed by one-way analysis of variance (ANOVA) with post hoc testing by the Newman-Keuls pairwise comparisons test between the experimental group and its controls (significance: $p<0.05$). ANOVA results are given as the value of the Fisher distribution F(x,y), where x is the number of degrees of freedom numerator and y is the total number of degrees of freedom denominator. Data are expressed as the mean ± s.e.m. with dots as individual values corresponding to a group of 40-50 flies analyzed together in a behavioral assay , to the response of a single recorded fly for lactate imaging and to one BODIPY stained brain for LD experiments, and to one mRNA extraction from heads of a group of 50 flies for RT-qPCR experiment.
Randomization	Flies were assigned to experimental groups based on genotypes.
Blinding	Data collection and analysis was not performed blind. Blinding is not applicable because the investigator who set up the experiment is the same person doing the analyses. However, each experiment was associated with proper controls, and sample were collected and analyzed under identical conditions.

Reporting for specific materials, systems and methods

We require information from authors about some types of materials, experimental systems and methods used in many studies. Here, indicate whether each material, system or method listed is relevant to your study. If you are not sure if a list item applies to your research, read the appropriate section before selecting a response.

Materials & experimental systems

n/a	Involved in the study	
☐	☒	Antibodies
☒	☐	Eukaryotic cell lines
☒	☐	Palaeontology and archaeology
☐	☒	Animals and other organisms
☒	☐	Human research participants
☒	☐	Clinical data
☒	☐	Dual use research of concern

Methods

n/a	Involved in the study	
☒	☐	ChIP-seq
☒	☐	Flow cytometry
☒	☐	MRI-based neuroimaging

Antibodies

Antibodies used	Primary antibodies:<br>RFP rabbit Clontech Cat#632496<br>nc82 mouse Developmental Studies Hybridoma Bank Cat#nc82<br>Wrapper mouse Developmental Studies Hybridoma Bank Cat#10D3<br>Secondary antibodies:<br>Alexa Fluor-488 anti-mouse Invitrogen #A11029<br>Alexa Fluor-594 anti-rabbit Invitrogen #A11037<br>Alexa Fluor-633 anti-mouse Invitrogen #A-21126
Validation	Validation for commercially available antibodies can be found using the links below:<br>RFP https://www.takarabio.com/learning-centers/gene-function/fluorescent-proteins/fluorescent-protein-antibody-citations/rfp-

antibody-citations
nc82 https://dshb.biology.uiowa.edu/nc82
Wrapper https://dshb.biology.uiowa.edu/10D3-anti-wrapper

Animals and other organisms

Policy information about studies involving animals; ARRIVE guidelines recommended for reporting animal research

Laboratory animals

Drosophila melanogaster flies were raised on standard food medium containing yeast, cornmeal and agar, on a 12h:12h light-dark cycle at 18°C with 60% humidity. The Canton-Special (CS) strain was used as the wild-type strain. All lines were out-crossed for at least three generations to flies carrying a CS wild-type background.
List of Drosophila strains:
Gal4 drivers:
pan neuronal driver elav-Gal4 line and tubulin-GAL80ts;elav-Gal4 line previsouly described in Silva et al., 2019 and pan glial driver Repo-Gal4 line described in Comas et al., 2004
tubulin-GAL80ts; VT30559-Gal4 previously described in Plaçais et al., 2017
tubulin-GAL80ts; Alrm-Gal4 and tubulin-GAL80ts; R54H02-Gal4 previously described in de Tredern et al., 2021
tubulin-GAL80ts; Repo-Gal4 and tubulin-GAL80ts; Mz0709-Gal4 constructed in this study using the Repo-Gal4 line or the Mz0709-Gal4 and the tubulin-GAL80ts line (referenced in the Methods section).
tubulin-GAL80ts; UAS-Dcr2, R54H02-Gal4 constructed in this study using the tubulin-GAL80ts; R54H02-Gal4 and the UAS-Dcr2 line (referenced in the Methods section).
Gal4 activity was released by transferring 0 – 2-day-old adult flies to 30°C for 2 days.
UAS-transgene lines:
from the Bloomington Drosophila Stock Center (BDSC): UAS-Dcr2 (BDSC: 24650), UAS-ACAT1 RNAi HMS03340 (BDSC:51785), UAS-Bmm RNAi JF01946 (BDSC:25926),UAS-CPT1 RNAi HMS00040 (BDSC:34066), UAS-HMGS RNAi HMC04928 (BDSC: 57738), UAS-AMPK RNAi JF01951 (BDSC:25931), UAS-AMPK RNAi HMC04979 (BDSC:57785) and UAS-mCD8::RFP (BDSC: 33219),
from the Vienna Drosophila Resource Center (VDRC): UAS-ACAT1 RNAi GD7132 (VDRC:v16099), UAS-Sln RNAi GD1940 (VDRC:v4607), UAS-Sln RNAi, KK104306 (VDRC: v109464), UAS-Bmm RNAi GD5139 (VDRC: v37877), UAS-CPT1 RNAi KK100935 (VDRC: v105400), UAS-Chk RNAi GD1829 (VDRC:v37139), UAS-HMGS RNAi KK107372 (VDRC: v108245) and mutant line: ChkMB04207 line (BDSC: 24296)
Reporter lines used in this study include CRIMIC Sln-T2A-Gal4 from BDSC (BDSC: 79274), and Chk-Gal4MI15450, provided by Dr. J. Sierralta.
The UAS-Laconic line was generated previously in our research group and used in Hudry et al. Cell. 2019.

All animals used in the study are F1 flies of 2-5 days old of mixed sexes except for imaging experiments (as detailed in the Methods) and for experiment with the Chaski mutant line MB04207 (Extended data 7b) in which only F1 females flies were used.

Wild animals

The study did not involve wild animal

Field-collected samples

The study did not involve samples collected from the field

Ethics oversight

No ethical approval or guidance was required since in this study we used Drosophila melanogaster.

Note that full information on the approval of the study protocol must also be provided in the manuscript.
